# Long-term stability of marine dissolved organic carbon emerges from a neutral network of compounds and microbes

**DOI:** 10.1038/s41598-019-54290-z

**Published:** 2019-11-28

**Authors:** A. Mentges, C. Feenders, C. Deutsch, B. Blasius, T. Dittmar

**Affiliations:** 1 Marine Geochemistry, Institute for Chemistry and Biology of the Marine Environment, University of Oldenburg, Oldenburg, Germany; 2 Mathematical Modeling, Institute for Chemistry and Biology of the Marine Environment, University of Oldenburg, Oldenburg, Germany; 3 School of Oceanography, University of Washington, Seattle, WA 98195 USA; 4 Department of Biology, University of Washington, Seattle, WA 98195 USA; 5 Helmholtz Institute for Functional Marine Biodiversity at the University of Oldenburg, Oldenburg, Germany

**Keywords:** Carbon cycle, Biogeochemistry

## Abstract

Dissolved organic carbon (DOC) is the main energy source for marine heterotrophic microorganisms, but a small fraction of DOC resists microbial degradation and accumulates in the ocean. The reason behind this recalcitrance is unknown. We test whether the long-term stability of DOC requires the existence of structurally refractory molecules, using a mechanistic model comprising a diverse network of microbe-substrate interactions. Model experiments reproduce three salient observations, even when all DOC compounds are equally degradable: (i) >15% of an initial DOC pulse resists degradation, but is consumed by microbes if concentrated, (ii) the modelled deep-sea DOC reaches stable concentrations of 30–40 mmolC/m^3^, and (iii) the mean age of deep-sea DOC is several times the age of deep water with a wide range from <100 to >10,000 years. We conclude that while structurally-recalcitrant molecules exist, they are not required in the model to explain either the amount or longevity of DOC.

## Introduction

Dissolved organic carbon (DOC) represents one of the Earth’s major carbon pools. It contains a similar amount of carbon as the atmosphere and exceeds the amount of carbon bound in marine biomass by more than two-hundred times^1^. DOC is mainly produced in the near-surface layers during primary production and grazing processes^2^. Other sources of marine DOC are dissolution from particles^2^, terrestrial and hydrothermal vent input^3^, and microbial production. Prokaryotes (bacteria and archaea) contribute to the DOC pool via release of capsular material, exopolymers, and hydrolytic enzymes^2^, as well as via mortality (e.g. “viral shunt”). Prokaryotes are also the main decomposers of DOC, although for some of the most recalcitrant forms of DOC very slow abiotic degradation in hydrothermal systems^2^ or possibly sorption to sinking particles^4^ may be the main removal mechanism. Mechanistic knowledge about DOC-microbe-interactions is crucial to understand the cycling and distribution of this active carbon reservoir.

DOC is conceptually divided into labile DOC, which is rapidly taken up by heterotrophic microbes, and the recalcitrant DOC reservoir, which has accumulated in the ocean (following the definition by Hansell^4^). As a consequence of its recalcitrance, the accumulated DOC reaches average radiocarbon ages of ~1,000–4,000 years in surface waters and ~3,000–6,000 years in the deep ocean^5^, indicating that it persists through several deep ocean mixing cycles of ~300–1,400 years each^6^. Behind these average radiocarbon ages, a large spectrum of ages is hidden: Follett *et al*.^7^ showed that DOC comprises a fraction of modern radiocarbon age, as well as DOC reaching radiocarbon ages of up to 12,000 years.

The surprising resistance of high concentrations of DOC to microbial degradation has been addressed by several hypotheses (e.g. Dittmar^8^). The prevalent notion is that the recalcitrant fraction of DOC has certain chemical properties, which prevent decomposition by microbes (“intrinsic stability hypothesis”). An alternative or additional explanation is given by the “dilution hypothesis”, that all compounds are labile, but exist in concentrations individually too low to sustain microbial populations but collectively form a large pool^9^. The dilution hypothesis found support in recent experimental and theoretical studies^10,11^, which sparked a vivid discussion of its plausibility^5,12–16^. To date, little is known about the substrate affinity of microbes towards DOC compounds^17^, therefore both the intrinsic stability of compounds and dilution limitation are possible scenarios.

Various models have been used to approach the unanswered questions regarding the stability and decomposition of dissolved organic matter (DOM), i.e. of dissolved organic carbon, nitrogen, and phosphorus. In existing DOM models, DOM stability is assumed to result from either intrinsic recalcitrance (RDOC_t_^18^) or dilution limitation (RDOC_c_^18^). Intrinsic recalcitrance is reflected in DOM models in the form of a DOM fraction unavailable to microbial degradation^19–22^, a fraction of DOC which is assumed to be less degradable^23–25^, or in distinct DOM pools with fixed life-times and no exchange between them^1,26^. Dilution limitation is incorporated into DOM models in the form of a lower concentration limit, below which microbial uptake of DOM is suppressed^14,22^.

The objective of our study was to numerically evaluate long-term (years to millennia) DOC stability and dynamics in a theoretical scenario of complete neutrality of compounds. We model DOC dynamics by neglecting any structural reactivity differences among compounds. We test whether this extreme scenario can lead to a realistic size and age of the DOC pool, despite the lack of any abiotic stabilization mechanism of DOC. In contrast to existing models, we model the dynamics of DOC and microbes without assuming intrinsic recalcitrance, and without a mathematical formulation of a concentration limit for microbial DOC uptake. The uptake of individual DOC constituents is slowed down at low concentrations, according to the well-established Michaelis-Menten kinetic (e.g. Polimene *et al*.^23^, Grégoire *et al*.^27^), but it is not suppressed completely, as long as the microbial population persists. In this regard, the model is different from the “dilution hypothesis”^10^, which proposes a fixed lower limit of bulk DOC below which no DOC uptake can take place. Our experiments are not designed or intended to prove or disprove the existence of structurally-recalcitrant molecules, which exist in the ocean for example in the form of black carbon^28,29^. The aim of this study is to explore the possible implications of a large diversity of equally reactive compounds on the size and age of the marine DOC pool.

We emphasize that simplification is one of the crucial steps in model formulation, allowing to identify causal relationships within complex systems by purposefully excluding most of the natural variety and focusing on selected key processes. Therefore, our model neglects abiotic production and removal processes, direct interaction among microbes, and higher trophic levels, for example. By representing intricate geochemical and ecological interactions in a simplified but mechanistic manner, our model helps to identify fundamental and emergent properties the DOC reservoir that serve to guide further observations and experiments.

**Figure 1:**
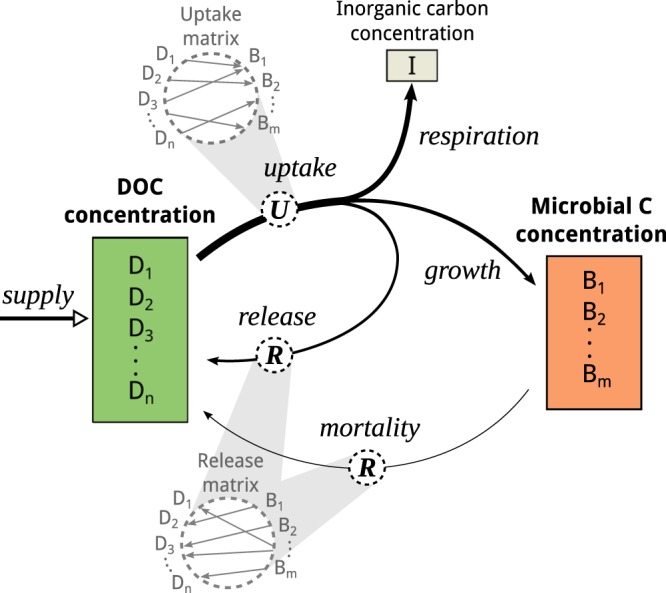
Scheme of the DOC-microbe-interaction model. The model predicts the carbon concentration of individual microbial units *B*_*i*_, individual DOC compound units *D*_*j*_, and the inorganic carbon pool *I* (note that due to the study focus the results for *I* are not shown). The arrows depict fluxes of carbon, where the width of the arrow indicates the strength of the flux. The carbon consumed is split among three pathways: respiration, microbial growth, and release of transformed compounds. The transformation of compounds is defined by the uptake and release matrices (dotted circles), which transfer carbon between pools. Each microbial unit takes up and releases a specific set of compounds. The supply of DOC represents an external source of DOC, e.g. from primary production.

We model the degradation and accumulation of marine DOC, assuming a large network of DOC compounds and microbes (Fig. 1). The microbes take up DOC, fix a fraction of that carbon into their biomass, respire a fraction of it to inorganic carbon (CO_2_), and release the remaining carbon as transformed DOC compounds back to the DOC pool. Microbes also contribute to the formation of DOC via lysis. New DOC is supplied from unspecified sources external to the microbe-resource network, which may include primary production, particle dissolution, or hydrothermal vents.

The model comprises a variety of “microbial units”, which each take up and release a specific set out of the modelled “DOC compound units”. A microbial unit is here defined as the group of heterotrophic bacteria or archaea that can take up the same groups of compounds. The DOC compounds are grouped hypothetically by structural properties, which govern uptake. Each microbial unit takes up and releases a specific set of compound units, forming a complex, bipartite network of DOC-microbe-interactions (see Material and Methods for an illustrative example). Assuming neutral reactivity, we neglect any reactivity differences among the DOC compounds. Accordingly, the uptake rate of a compound depends exclusively on its concentration (Michaelis-Menten kinetic). At low concentrations, the uptake is slower, but there is no physiological lower limit for microbial DOC uptake. Published estimates served as a basis for the parameterization of e.g. the uptake and mortality rate of microbes (for details see Materials and Methods). Unfortunately, the exact values of the model parameters are poorly constrained for marine organisms, especially for the deep sea and for archaea. To address this uncertainty, we tested the sensitivity of model results using a range of model parameters.

We aim to reproduce essential features of DOC in the ocean with our conceptual model. The model should thus be in agreement with the following observations concerning DOC: (i) microheterotrophs form recalcitrant DOC^30–33^, which is (ii) taken up if concentrated^10^, (iii) DOC concentrations range between 30–80 mmolC/m^3^ in the ocean^1,34^, and (iv) DOC shows a broad spectrum of radiocarbon ages in the deep sea from 10 to ~10,000 years^7^. Targets of our numerical model are not short-term dynamics of DOC turnover, e.g. during and after a phytoplankton bloom. On these time scales, the affinity of microbial taxa to specific substrate compounds is clearly important. For the purpose of our study we purposefully refrain from ascribing different affinities to different DOC compounds, in order to test the explanatory power of a model with neutral reactivity.

## Results and Discussion

### Formation of recalcitrant DOC in incubation experiments

**Figure 2:**
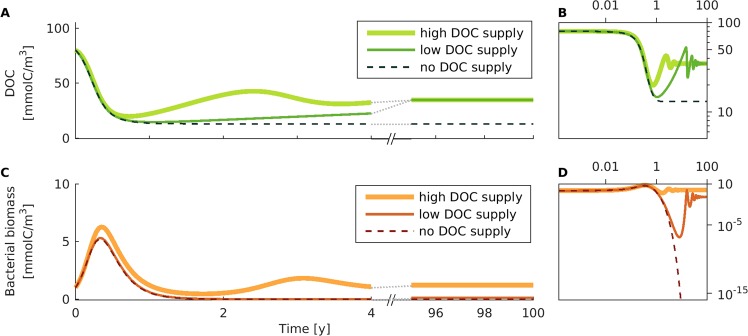
Formation of recalcitrant DOC in incubation experiments. (**A**) Total DOC concentration and (**C**) total microbial biomass is shown over simulation time, for three levels of external DOC supply: high (bold lines), low (thin lines), and no supply (dashed lines). Note that in panels (A,C) the horizontal axis is broken after five years, continuing to show years 95–100. (**B**,**D**) Full time-series is shown on a double-logarithmic scale.

The amount of (apparently) recalcitrant DOC is determined after 100 simulation years of degradation of an initial DOC pulse (Fig. 2). This is done for three different scenarios: (a) no supply of DOC compounds from primary production, representing a bottle experiment, (b) low supply of DOC, representing low nutrient regions, and (c) high supply of DOC, representing bloom conditions.

DOC and microbial biomass concentrations are in the range of observed values for each of the three scenarios (Fig. 2). Without any DOC supply, the percentage of recalcitrant DOC (~16% of initially provided DOC, corresponding to 13 mmolC/m^3^) is comparable to experimental results (5–10% for simple substrates^30–32^; 35–80% for more diverse substrates^32,33^). The model is designed to study the long-term stability of DOC in the ocean, and it is parameterized according to published values for deep-sea microbial communities. As such, it overestimates the time scale of initial DOC consumption (~200 simulated days, reported degradation time-scales are ~3–21 days^30–33^). Due to the limited amount of carbon available in the scenario of no supply, the microbial community continuously declines in biomass and becomes vanishingly small after about one year.

For low and high DOC supply rates, the DOC concentration after 100 simulation years is close to values observed in the deep Pacific Ocean (~43% of initial DOC, corresponding to 35 mmolC/m^3^ ^1^). The final microbial biomass is close to values observed for free-living bacteria in the bathypelagic ocean (0.12 mmolC/m^3^ ^35^), for the low-supply-scenario and in the range of values observed for the epipelagic ocean (1.21 mmolC/m^3^ ^35^), for the high-supply-scenario (see Supplement Fig. S1 for the concentration distribution of compounds at the end of the simulation).

Our model shows the formation of an (apparently) recalcitrant DOC pool from bioavailable DOC. At the end of the virtual incubations, a significant fraction of the initial DOC concentration is left, independent of the supply rate (Fig. 2, high or low DOC supply). The microbial production of a heterogeneous mixture of recalcitrant DOC compounds has been observed in laboratory experiments^30,31^.

Recalcitrance of DOC in the model results from two different mechanisms: (a) if there is no supply of DOC, the microbes go almost extinct after the initial bloom and thus “leave behind” a residual concentration of DOC, and (b) if there is supply of DOC, microbes maintain a stable population on the long-term and therefore the DOC concentration reaches a steady-state where uptake, release, and supply are balanced (see double-logarithmic inlays in Fig. 2).

On a time scale of years to decades, the amount of recalcitrant DOC depends on the supply rate (Fig. 2). The reported accumulation of DOC in the surface layer of the Atlantic Ocean depends on the rate of New Primary Production^36^. Our model is in agreement with this observation: on short time scales (<50 years, i.e. shorter than the residence time of surface water), DOC concentrations increase with its supply rate. On longer time-scales, however, the amount of (apparently) recalcitrant DOC does not depend on the supply rate (Fig. 2A,C). In the future centuries, increased levels of CO_2_ in the atmosphere might enhance the algal production of DOM^37,38^, and thus the supply rate of DOC. Experiments showed that there is no significant change in DOC concentration under increased CO_2_ levels^39^. Our model supports this observation: increased primary production, represented by elevated supply rates, does not affect long-term DOC concentrations. However, it increases the microbial biomass by orders of magnitude (Fig. 2B,C, and Supplement Fig. S2J). The “additional” DOC from increased primary production is thus partially stored in the biomass of consumers.

### Size of the DOC reservoir: A sensitivity analysis

**Figure 3:**
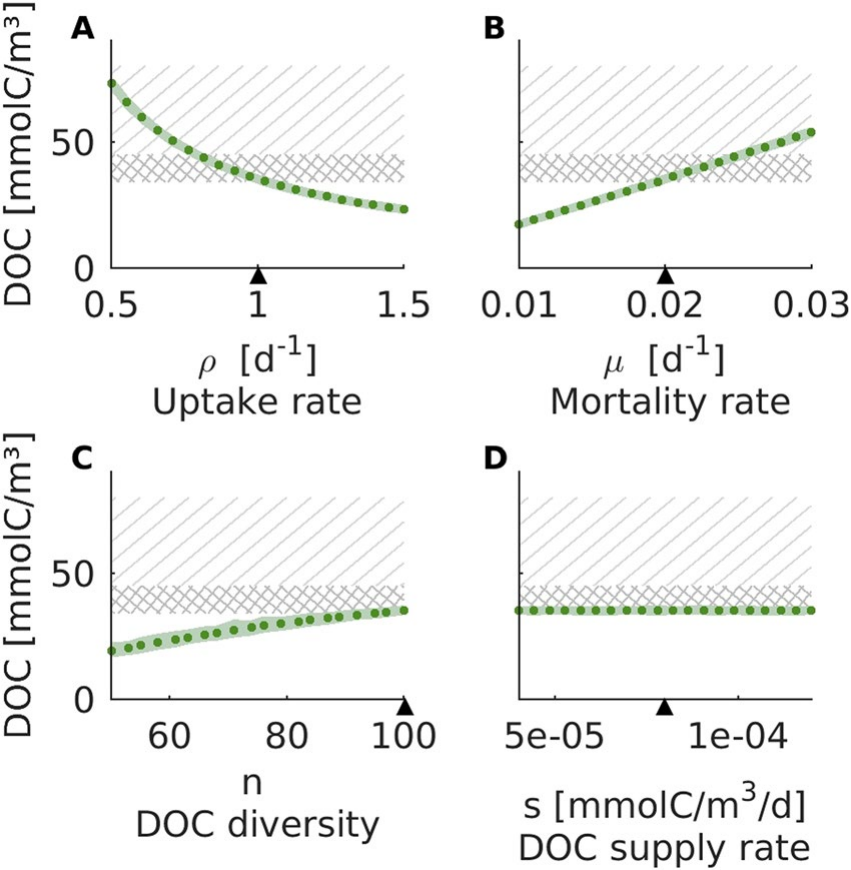
Size of the DOC reservoir. The total concentration of DOC after 20,000 simulation years for varying parameter values: (**A**) the microbial uptake rate ρ, (**B**) the microbial mortality rate µ, (**C**) the number of DOC units *n*, and (**D**) the total supply rate of DOC *s* (see Supplement, Fig. S4 for the remaining parameters). Each simulation was repeated 50 times, the green dots indicate the mean, the light green area represents the minimum and maximum concentrations from the 50 runs. The black triangle indicates the default value of the parameter. The hatched region indicates the range of DOC concentrations typically observed in the surface ocean, the cross-hatched region indicates typical deep sea DOC values^1,34^. For more scenarios we refer to the Supplementary Material.

To assess the sensitivity of the DOC reservoir to model parameters, we determine the steady-state DOC concentration for a range of parameter values (Fig. 3, see supplement for a sensitivity analysis of microbial biomass, mean DOC age, and other model parameters, Fig. S2, Fig. S3, Fig. S4). While one parameter is varied, the others are kept at their default value. Overall, the size of the DOC reservoir changes by a factor of about 0.5–1.5 across all tested parameter ranges. The model results are thus robust to moderate variation of the parameters. Large deviations only occur when varying parameters by orders of magnitude. The model predicts steady-state DOC concentrations between 17–74 mmolC/m^3^, covering the range of marine DOC observations^1,34^.

Increasing parameters that fuel microbial growth (i.e. maximum uptake rate ρ, the proportion of substrates taken up per microbe *n*_*U*_*/n*, and the microbial growth efficiency) decreases the size of the DOC reservoir (Fig. 3A, and Supplement Fig. S4). In contrast, increasing parameters that constrain microbial biomass (i.e. mortality rate µ and half-saturation constant κ) increases the size of the DOC reservoir (Fig. 3B, and Supplement Fig. S4).

The size of the DOC reservoir also increases with the diversity of DOC compounds (number of DOC compound units *n*, Fig. 3C). The microbial community reduces the concentration of a DOC compound until the energetic costs for survival and maintenance (i.e. respiration, mortality) exceed the amount of carbon gained through uptake of this compound unit (according to resource competition theory^40^). Each compound is therefore left over at its limiting concentration (i.e. the equilibrium DOC level D*). The higher the DOC diversity, the more compounds at their individual limiting concentrations add up to a larger total DOC pool.

It might seem surprising that the supply rate of DOC does not affect the steady-state DOC concentration (Fig. 3D). However, because DOC is supplied at a constant rate, microbes adapt to this level of carbon available and are able to maintain higher levels of biomass (see Supplement Fig. S2F). Hence, the supplied carbon is converted to microbial biomass, leaving the steady-state DOC concentration unaltered. Note that while this is true on long time-scales (millennia, or steady-state, respectively), on short time-scales (years to decades) the size of the DOC reservoir depends on the amount of supply from primary production (Fig. 2A).

We identified the number of compound units *n*, the number of compounds taken up per microbial unit *n*_*U*_, and the half-saturation constant κ as important factors for the steady-state DOC concentration, while being the least constrained by published referenced values. An increase in the number of compound units *n* can be “compensated” (i.e. the steady-state DOC concentration can be preserved) by either an increase in the number of taken-up compound units per microbial unit *n*_*U*_ by the same factor, or a proportional decrease in the half-saturation constant κ (see Supplement Fig. S5C).

For a general overview of the sensitivity of the DOC concentration to parameter variations at steady-state we take advantage of the mean field theory. According to this theory, our interactive network model collapses into a simple equation if total neutrality is assumed (see supplement for details). In that case, the size of the DOC reservoir in steady-state D* can be estimated as

1 $${D}^{\ast }=\frac{\mu \kappa n}{\eta \rho {n}_{U}}.$$

This equation summarizes the influence of model parameters on the long-term DOC concentration D*. It is proportional to mortality rate *µ*, the half-saturation constant of DOC uptake *κ*, and the diversity of DOC compounds *n*. The long-term DOC concentration is constrained by the microbial growth efficiency *η*, the maximum uptake rate *ρ*, and the number of compound units consumed per microbial unit *n*_*U*_.

Note that the equilibrium DOC level D* is not a parameter of the model, but a consequence of the basic ecological processes that were modelled. This simple equation describes the long-term DOC concentration based on the turnover of an average DOC compound by an average microbial unit. For other, more detailed model outcomes, e.g. the distribution of concentration across compounds and microbes, or the age distribution of DOC, the full network model is required. The equilibrium concentration D*, which can be derived without simulation, is a minimum DOC estimate. In the case of non-neutral DOC uptake (reactivity classes in alternative model set-up, see supplement), the final DOC concentration is equal to or higher than D*. Substrate diversity in relation to microbial uptake capabilities (*n*/*n*_*U*_) emerges as one of the key drivers that determine the size of the DOC pool in the ocean. At a fixed microbial uptake capability (fixed number of DOC compound units taken up per bacterial unit *n*_*U*_), an increase in DOC compound diversity implies an increase in the long-term DOC concentration D*.

### Millennial scale stability of DOC

**Figure 4:**
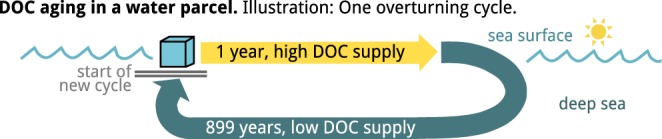
Illustration of millennial scale DOC stability simulation. The aging of DOC is simulated in a water parcel moving along the ocean circulation. The journey of the water parcel in 25 overturning cycles is simulated by applying a varying DOC supply: at the beginning of each cycle, one year of surface conditions is simulated in the form of high supply of DOC, whereas for the following 899 years low supply rates of DOC simulate deep-sea conditions. The variations in total supply rate affect the total DOC concentration and the concentration-weighted mean DOC age, for results see Fig. 5.

To approximate the persistence of DOC in the ocean, the radiocarbon age of DOC compounds is simulated in an isolated, Lagrangian water parcel moving along the deep oceanic circulation (Fig. 4). The total supply rate of DOC is variable over time, to mimic the changes between the rapid DOC supply in surface waters and the much slower DOC supply in the deep-sea. To reflect the selective production of DOC compounds by an algal bloom, only a subset of compounds is supplied (3 out of 100, value chosen to represent a scenario where most compounds are exclusively produced by microbial consumers).

**Figure 5:**
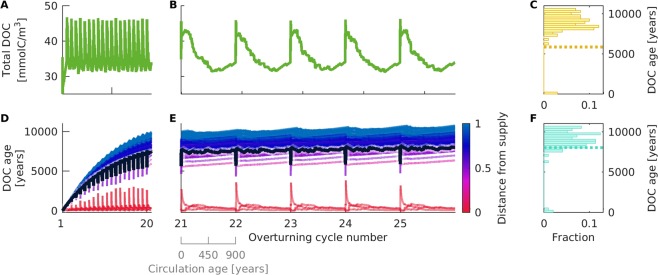
Millennial scale stability of DOC. The variations in total supply rate along the simulated Lagrangian ocean circulation (see Fig. 4) influence (**A**, **B**) the total DOC concentration and (**D**,**E**) the concentration-weighted mean DOC age. The bold, black line indicates the concentration-weighted mean of the average compound ages. The thin coloured lines show the mean compound age of the individual DOC compound units. Their colour indicates the proportion of microbially-reworked to primary-produced compounds: red compounds are directly supplied via primary production; blue compounds are exclusively microbially reworked. The left column of panels shows the first 20 overturning cycles, during which the system approaches a dynamic equilibrium. The right column shows the last five overturning cycles of the equilibrated system in detail. The distribution of DOC ages across compounds in the last overturning cycle is shown for the (**C**) surface and deep (**F**) supply regime. The dotted lines indicate the concentration-weighted mean age.

The concentration of DOC and microbial biomass in the overturning cycles is close to observations. During the surface-supply-regime, the total DOC concentration corresponds to values observed in most of the surface and mesopelagic ocean^1,34^ (Fig. 5A,B; see Supplement Fig. S6 for biomass). During the transition from the surface to the deep-sea supply regime, the DOC concentration slowly decreases to values representative for North Pacific deep waters^1^. The dynamics of DOC are slightly different among the overturning cycles, indicating that the duration of one overturning cycle did not allow for complete equilibration of the system within one cycle.

The mean DOC age increases over time, until it equilibrates after about 20 cycles at an age of ~8,200 years (Fig. 5D,E). The DOC age temporarily decreases at the start of each new overturning cycle, as the water parcel receives high rates of new DOC with zero ‘age’. The difference in the age of surface DOC (~5,800 years, Fig. 5C) and deep sea DOC (~8,000 years, Fig. 5F) is similar to the age difference observed in the ocean (~1,000–3,000 years^5^). The age of DOC compounds in the model ranges from less than hundred to several thousand years (~80–11,000 years). The age spectrum of modelled DOC compounds is thus in agreement with observations of marine DOC, which shows a broad spectrum of radiocarbon ages from modern up to 12,000 years^3,7^.

Two groups of compounds can be distinguished by their mean ages (Fig. 5C,E,F): compounds that reach ages of ~80–280 years, i.e. centennial compounds (depicted in red in Fig. 5E), and compounds that reach ages of 6,000–11,000 years, i.e. millennial compounds (depicted in blue in Fig. 5E). The centennial compounds comprise those three compound groups that are supplied from external sources such as primary production. The millennial compounds comprise all other compounds, which are exclusively produced by microbes, at much lower rates. The modelled age spectrum thus emerges from differences in the supply rate of compounds (i.e. their rate of autotrophic production). Note that our simulated microbes do not distinguish between the centennial and millennial compounds during uptake or release. The uptake and release network is constructed randomly, without any preference for the two compound groups. A microbe in the model could be able to take up centennial along with millennial compounds, depending on the random outcome of the network. The bi-modal age distribution in the simulation (Fig. 5C,F) is consistent with observations^7^. The relative width of the two age peaks and their distinctiveness is set by the difference in the supply rate of the respective compounds.

The more microbial reworking is needed to form a compound from algal-produced DOC, the older it becomes. In this sense, our model is in agreement with the processes termed the “microbial carbon pump”: DOC with high mean ages is produced by microbes^41^. The variation in ages within the two age classes is explained by the uptake and release within the network of compounds and degrading microbes. Within the microbial compounds (average age of ~8,000 years), for example, some compounds are relatively younger, showing ages of only ~6,000 years. Those compounds are direct release products of microheterotrophs which consume fresh, algal DOC. The age of a compound is thus a consequence of its supply rate and its position in the network of uptake and release preferences. This mechanism could explain observed DOC spectra additional to the traditional view that the observed age spectrum of DOC compounds arises from differences in intrinsic reactivity of DOC compounds^42^.

Age fractions of observed marine DOC show distinct structural properties: Fresh DOC compounds are larger, have a lower C:N ratio, and a higher percentage of common biochemicals than aged DOC compounds^42,43^. This co-occurrence of structural and age differences does not necessarily require the assumption of intrinsic compound stability. If structurally different compound classes differ in production rates, the structural signature will be reflected in the age fractions. Hence, if we assume a general decrease in size from highly-produced compounds (e.g. algal DOM) to low-produced compounds (e.g. hydrothermal DOM^44^), then aged, modelled compounds will be smaller in molecular size than young compounds. Indeed, algal DOM has been shown to be relatively large, whereas hydrothermally altered DOM shows decreased molecular mass. Accordingly, the model results can be reconciled with the size-reactivity-continuum^43^.

### Implications for DOC stability

#### The mechanism behind the longevity of DOC in the model

Although we assume bioavailability of all compounds, the model predicts long-lived fractions of DOC (Fig. 5). Usually, it is assumed that microbially-produced DOC in the ocean persists over millennia, because bacteria alter its structure, making it intrinsically stable^16,42^ (see Supplement Fig. S7 for a comparison to Shen & Benner’s experiment). However, in our model, microheterotrophs cannot alter the reactivity of DOC compounds. Instead, microbes exclusively re-distribute the carbon among the compounds (through the network of uptake and release) and reduce DOC concentration (through respiration).

Ultimately, the longevity of DOC in our model can be explained by the limitation of microbial growth by low DOC concentrations. This corresponds to the well-known resource competition theory of Tilman: Analogous to the R* value, that gives “the levels to which each competitor can reduce a single limiting resource”^40^, we propose a D* value, the minimum DOC concentration that can be reached by biological degradation. For a scenario of neutrality, D* can be estimated from Eq. 1, otherwise the full network model is required. D* is reached when the growth of the microheterotrophs balances their mortality, and when the production of DOC (i.e. the sum of supply, release, and lysis) balances its biological degradation. In this steady-state, DOC concentrations and biomasses equilibrate, such that net changes become zero. This equilibrium DOC level does not allow for further microbial growth and biomass increase; hence DOC is (apparently) recalcitrant.

If the equilibrium DOC level D* is surpassed via supply of DOC compounds from an external source, the formerly recalcitrant DOC allows for microbial growth^10^ (see Supplement Fig. S8). Through this process, recalcitrant deep-sea DOC can be microbially removed near the ocean surface, as reported e.g. by Cherrier *et al*.^45^ (the authors of this study suggest photodegradation causes this removal, here we offer an alternative explanation). According to the notation of Jiao *et al*.^18^, the equilibrium DOC level D* would correspond to the *RDOC*_*c*_, defined as “compounds that may be inaccessible to microbes due to their extremely low concentration“. Assumptions about *RDOC*_*t*_ (“compounds that are resistant to microbial consumption in certain environments, but subject to further cleaving and decomposition when the situation changes”^18^) are not required to form a recalcitrant carbon pool at concentrations close to observed values for experiments and the deep sea in our model.

#### Intrinsic stability: An alternative model set-up

Additional to our neutral model set-up, we implemented an alternative model set-up comprising the five traditional reactivity classes of DOC (labile, semi-labile, semi-refractory, refractory, and ultra-refractory^4^). In this alternative model set-up, intrinsic reactivity differences are implemented by successively reducing the maximum uptake rate by an order of magnitude for each less reactive DOC class (Supplementary material, Fig. S9). The supply of DOC is restricted to the labile DOC class, i.e. recalcitrant DOC is exclusively microbially-produced. In this alternative model set-up, DOC concentration does not reach a steady-state, but increases due to ongoing accumulation of the least reactive DOC classes (i.e. refractory and ultra-refractory DOC). Applied to the ocean, this would imply an increase of DOC concentrations with each ocean overturning. To achieve an equilibrium DOC level in this “intrinsic stability model”, additional abiotic removal process to microbial consumption would have to be postulated, e.g. adsorption to sinking particles or photodegradation in the sunlit ocean^4^.

### Main findings

We use a network model of DOC-microbe-interactions to predict long-term DOC behaviour. In our model all DOC compounds are equally bioavailable. Our numerical model is in agreement with observed characteristics of marine DOC (Table 1).

**Table 1:** Overview of observed DOC characteristics and corresponding model behaviour.

	Observed characteristics	Model behaviour
*Recalcitrance of DOC*	>5% of DOC is left in microbial degradation experiments^30–33^.	>10% of DOC is left after 100 simulation years (Fig. 2).
*Size of the DOC reservoir*	DOC shows concentrations of 30–80 mmolC/m^3^ in the ocean^1,34^.	DOC concentrations range between 17–74 mmolC/m^3^ for reasonable variations of parameters (Fig. 3).
*Millennial scale stability*	DOC compounds reach ages between modern and >10,000 years^7^. The mean DOC age differs by about ~1,000–3,000 years between surface and deep sea^5^.	DOC compounds reach ages between ~80 to 11,000 years (Fig. 5). The mean DOC age differs by 2,200 years between surface and deep-sea conditions (Fig. 5C,F).

In our model, a significant fraction of the bioavailable DOC resists microbial degradation, forming an apparently recalcitrant DOC pool. A fundamental microbiological principle prevents further degradation of DOC at low concentrations: microbial growth ceases when the concentration-dependent uptake of DOC balances the concentration-independent microbial carbon loss via mortality or maintenance, resulting in an equilibrium DOC level (D*). Consequently, the apparently recalcitrant DOC becomes available for microbial degradation, if concentrated. Under the assumption of complete neutrality, D* can be estimated through an extended version of the Michaelis-Menten concept where substrate diversity and microbial capabilities (*n*/*n*_*U*_) play a central role (Eq. 1).

We show that the size of the modelled DOC reservoir corresponds to observations in the deep ocean. Individual, equally bioavailable DOC compounds reach radiocarbon ages between modern and several thousand years. The age of DOC compounds in our model is not determined by intrinsic reactivity, but by their position in the network of microbial uptake and release, and, most importantly, by their rate of supply from primary production. Overall, our results indicate that the long-term stability of marine DOC emerges from basic physiological properties of the individual members of the degrading microbial community, and the resulting interactions between the individual microbial members through substrate units. As such, DOM stability is possibly neither a direct consequence of intrinsic recalcitrance, nor simply due to a fixed dilution threshold. The aim of this modelling study was not to prove or disprove the existence of intrinsically recalcitrant features in DOC, but to show that assuming neutral, concentration-limited uptake, a realistic size and age of the DOC reservoir can be reached.

## Methods

### Model equations

We consider *m* taxonomic units of microheterotrophs *B*_*i*_ (i = 1, 2, …, *m*) and *n* substrate units of DOC compounds *D*_*j*_ (j = 1, 2, …, *n*). Our model resolves the carbon concentration (in mmolC/m^3^) of these microbial units and DOC compounds in a mixed culture. Microbial units are measured in units of carbon to avoid stoichiometric conversion between the state variables. Modelled processes are summarized in Fig. 1. The model is composed of a set of ordinary differential equations, one for each individual microbial unit *B*_*i*_ and one for each DOC compound unit *D*_*j*_ (see Supplementary Material Equation (4) for the inorganic carbon pool *I*, equations (5–13) for the radiocarbon age, and Table S1 for a list of symbols and abbreviations):

2 $${\dot{B}}_{i}=\eta \mathop{\sum }\limits_{k=1}^{n}\,{{\bf{U}}}_{ik}\frac{\rho {D}_{k}}{{D}_{k}+\kappa }{B}_{i}-\mu {B}_{i}$$

3 $${\dot{D}}_{j}=\mathop{\underbrace{-\mathop{\sum }\limits_{i=1}^{m}\,{{\bf{U}}}_{ij}\frac{\rho {D}_{j}}{{D}_{j}+\kappa }{B}_{i}}}\limits_{{\rm{Bacterial}}\,{\rm{DOC}}\,{\rm{uptake}}}+\mathop{\underbrace{\mathop{\sum }\limits_{i=1}^{m}\,{{\bf{R}}}_{ij}\,\mu {B}_{i}}}\limits_{{\rm{Mortality}}}+\mathop{\underbrace{\beta (1-\eta )\mathop{\sum }\limits_{i=1}^{m}\,{{\bf{R}}}_{ij}\mathop{\sum }\limits_{k=1}^{n}\,{{\bf{U}}}_{ik}\frac{\rho {D}_{k}}{{D}_{k}+\kappa }{B}_{i}}}\limits_{{\rm{Bacterial}}\,{\rm{release}}\,{\rm{of}}\,{\rm{transformed}}\,{\rm{DOC}}}+\mathop{\underbrace{{s}_{j}}}\limits_{{\rm{Supply}}}$$

From the DOC uptake, a fraction η is fixed into microbial biomass, the fraction β(1 − η) is released back to the DOC pool as transformed compounds, and the remaining carbon is permanently transferred to the inorganic carbon pool (i.e. microbial respiration). DOC uptake is described according to the Michaelis-Menten kinetic, with the maximum uptake rate ρ and the half-saturation constant к. We assume that each substrate unit is concentration-limited individually. This means, if unit A is present at high concentrations and unit B at low concentrations, the former is taken up at high rates, while the uptake of the latter is concentration limited. Mortality of microbes is proportional to mortality rate μ and contributes to the formation of transformed DOC compounds. New DOC is supplied at the rate *s* from primary production (where the total supply rate *s* = ∑*s*_*j*_ is the sum of the supply rates for individual compounds *s*_*j*_). The uptake matrix ***U*** = (***U***_*ij*_) defines the uptake preferences of each microbial unit *i*, its entries ***U***_*ij*_ specify whether a microbial unit can take up a compound unit *j* (***U***_*ij*_ > 0). The release matrix ***R*** = (***R***_*ij*_) defines a specific set of release products for each microbial unit, as well as the partitioning of carbon among the release products. Its entries ***R***_*ij*_ specify the fraction of carbon released per compound unit. Together, ***U*** and ***R*** form a “transformation network”, as the taken-up carbon is re-distributed among DOC compounds for release. Altogether, the total microbial biomass, the total DOC concentration, and the inorganic carbon concentration (Σ*B*_*i*_, Σ*D*_*j*_, *I*) form a mass-conserving carbon pool.

### How the model works: an illustrative model set-up

**Figure 6:**
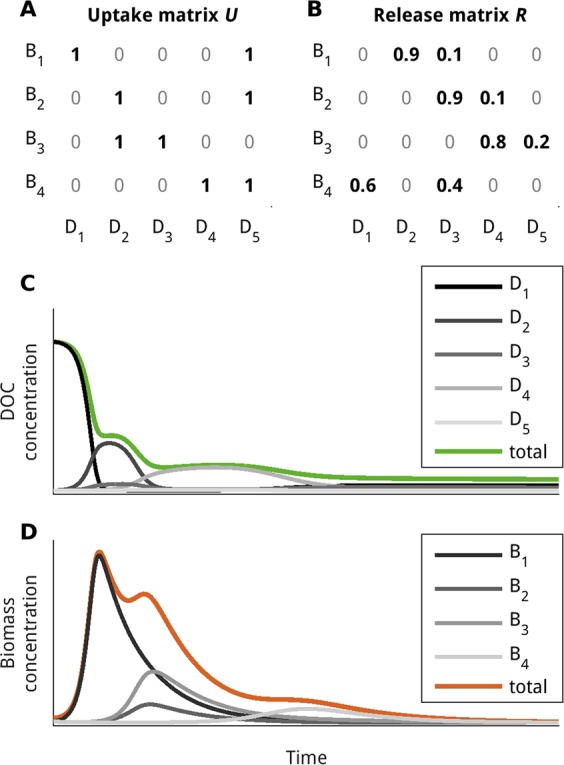
Illustration of the DOC decomposition model: a toy model set-up. The transformation network of DOC is defined by two matrices, (**A**) the uptake matrix **U**, and (**B**) the release matrix **R**. The model predicts (**C**) the concentration of individual DOC compound units *D*_1_,…, *D*_*n*_ and (**D**) the biomass of each microbial unit *B*_1_,…, *B*_*m*_ over time (here for simplicity only n = 5 and m = 4 groups are shown; see Supplement Fig. S10 for a corresponding representation with higher microbial and chemical diversity).

To illustrate the basic model behaviour, we present a toy set-up, with a small number of microbial and compound units (Fig. 6). Initially, the DOC compound unit *D*_1_ is provided as the only substrate (Fig. 6C). Initially, biomass of all microbial units is very low (Fig. 6D). The microbial unit *B*_1_ takes up the initially provided compound unit *D*_1_ (according to the uptake matrix in Fig. 6A, row 1, column 1), and transforms it into DOC compound units *D*_2_ and *D*_3_ (in a ratio of 9:1, according to the release matrix in Fig. 6B, row 1, column 2 and 3). The microbial units *B*_2_ and *B*_3_ start to grow on the newly produced compound units. *B*_3_ reaches a higher maximum biomass than *B*_2_, because it is able to grow on both compound units *D*_2_ and *D*_3_. The microbial unit *B*_3_ produces the compound units *D*_4_ and *D*_5_, which serves as a substrate for the last blooming microbial unit *B*_4_. This microbial unit releases carbon in the form of *D*_3_ (40%) and the initially provided compound unit *D*_1_ (60%), thereby closing the cycle of DOC re-working. The compound unit *D*_5_ remains at concentrations near zero throughout the virtual incubation, because it is produced only at 20% by a single microbial unit (*B*_3_), and it is taken up by all other three microbial units (*B*_1_, *B*_2_, *B*_4_). Due to the respiration of organic carbon to inorganic carbon (time series not shown) the total concentration of DOC declines over time. There is no supply of DOC in this virtual incubation.

This simulated initial DOC decomposition qualitatively matches observations. It has been shown experimentally that a sequence of microbial groups with distinct functional and transporter profiles occur during decomposition of algal-derived organic matter; as the substrates become available successively and enable different ecological niches to be filled^46^.

### Default parameterization

The model parameters were chosen according to published reference values, if available (Table 2). Note that some of the parameters (e.g. the total number of compound units *n*, the half-saturation constant κ, and the number of compound units taken up per microbial unit *n*_*U*_) are not well-constrained and the chosen parameter set has a high level of uncertainty. To account for this, we tested the sensitivity of steady-state total DOC, microbial biomass, and mean DOC age (see Fig. 3 and Supplement Figs. S2, S3) for each parameter. Microbial traits are assumed to be equal, apart from the uptake- and release abilities, i.e. all microbial units are assigned the same mortality rate, maximum uptake rate, half-saturation constant, and they take up and release the same number of compound units.

**Table 2:** Default parameter set.

Parameter	Value	Unit	Description
*m*	30		Total number of microbial units
*n*	100		Total number of compound units
*n*_*U*_	3		Number of compound units taken up per microbial unit
*n*_*R*_	30		Number of compound units released per microbial unit
η	0.20*		Fraction of uptake converted to microbial biomass
β(1 − η)	0.14^†^		Fraction of uptake released to DOC
ρ	1^‡^	d^−1^	Maximum microbial uptake rate
κ	10^‡^	mmolC/m^3^	Half-saturation constant of microbial uptake
μ	0.02^§^	d^−1^	Microbial mortality rate
*s*	0.08^||^	mmolC/m^3^/d	Supply of DOC in the euphotic zone (e.g. from primary production)

*^50^, †^30^, ‡^52^, §^53^, || estimated based on Hansell^4^.

#### Number of compounds, microbial units, substrates, and release products (*n, m, n*_*U*_, *n*_*R*_)

Marine microbes are highly diverse: Huber *et al*.^47^ estimated the presence of >35,000 marine operational taxonomic units. The molecular diversity of DOC compounds is also high: Zark *et al*.^48^ estimated that >100,000 different dissolved organic matter compounds exist in seawater. To increase computational efficiency, the diversity of both groups is reduced for numerical simulations. However, the diversity ratio (0.35: 1) is maintained by grouping marine microbes to a total of 35 microbial units, and DOC compounds to 100 compound units, respectively. Note that we define the compound units from a bacterial perspective: all compounds that can be taken up by the same physiological uptake mechanism (e.g. transporter proteins) are grouped together, i.e. in our model molecular structural details only matter if they are relevant in a physiological context. As published estimates are missing, we assume that a microbial unit can take up 3% of the compound units, and releases 30% of the compound units, reflecting the observation that organic matter is diversified by microbial degradation^31,49^. Note that the absolute numbers of *m*, *n*, *n*_*U*_, and *n*_*R*_ are not crucial in the context of the model, as long as their proportion and their relation with the uptake parameters is considered: the steady-state DOC concentration is independent of the number of microbial units (Fig. S5A), but is influenced strongly by the ratio *n*_*U*_*/n* (Fig. S5B) and the half-saturation constant κ (Fig. S5C, see also discussion of size of DOC reservoir). For example, the diversity of DOC and microbes could be increased proportionally by an order of magnitude (*m* = 350, *n* = 1050, *n*_*U*_ = 30), however, this would only increase the size of the uptake- and release network, and leave the long-term DOC concentration D* unaffected (see Eq. (1)).

#### Fractionation of uptake to biomass, release, and respiration (η, β)

We assume that the amount of carbon taken up by a microbe is split across three different pathways: the fraction η is assigned to microbial growth, the fraction β(1 − η) is released as transformed DOC, and the remaining fraction (1 − η)(1 − β) is respired (i.e. the carbon is permanently assigned to the inorganic carbon pool). Estimates from experimental studies are available for microbial growth efficiency, which we use to choose η (BGE = 0.09–0.33^50,51^). Note that productivity measurements are unlikely to record the released material, thus underestimating growth efficiency^50^ and potentially biasing the estimate. The fraction of carbon uptake that is released β(1 − η) was chosen based on observations by Ogawa *et al*.^30^, who found that 15% of taken up glucose was released as DOC after 2 days of incubation in experiments, and 13% for glutamate. The microbial growth efficiency could be reduced to account for additional energy required for the degradation of high molecular mass compounds via extracellular hydrolysis^17^. A reduction of the growth efficiency associated with the uptake of a fraction of DOC compounds by 10% would increase the long-term equilibrium concentration of this fraction by 10% (as given in Eq. (1)).

#### Microbial uptake and mortality (ρ, κ, μ)

Estimates for uptake parameters representative for the highly diverse microbial community of the global oceans are lacking to date, therefore uptake parameters were based on laboratory estimates of a facultatively oligotrophic marine ultramicrobacterium growing on either alanine or glucose in the absence of primary producers and grazers, yielding a maximum uptake rate ρ = 0.96–3.60 d^−1^ and a half-saturation constant κ = 6–450 mmolC/m^3^ ^52^. The model results are qualitatively robust towards variation of these parameters by a factor of 0.5 to 1.5 (Fig. 3A for uptake rate, Fig. S4D for half-saturation constant). As the published estimates vary by nearly two orders of magnitude, the half-saturation constant κ is not well-constrained. Changes in the value of κ can be balanced by proportional changes in the ratio of compounds taken up per microbial unit to the total DOC diversity *n*_*U*_*/n* (see Supplementary Figure S5). Note that this parameter choice likely overestimates microbial DOC consumption compared to oceanic conditions, yielding a conservative minimum estimate of the DOC reservoir size. It should also be noted that if uptake rates were assumed different among components, their individual long-term concentration would change accordingly, whereas the total DOC concentration would rather depend on the average over consumption rates. The mortality of microbes is assumed linearly proportional to their biomass, with a mortality rate μ = 0.02 d^−1^. Laboratory experiments with planktonic microbes showed specific mortality rates of 0.009–0.025 d^−1^ ^53^ in the absence of grazers, similar to rates used in other modelling studies (0.031–0.05 d^−1^ ^19,23^).

#### Supply of DOC (*s*)

Based on a labile DOC production in the ocean of 25 PgC/y = 5.71 × 10^15^ mmolC/d^4^, euphotic zone depth of 200 m, and global ocean surface area of 3.62 × 108 km^2 ^^54^, the total supply rate of DOC *s* in the surface layer is assumed to be 3.45 × 10^−7^ PgC km^−3^ y^−1^ = 0.08 mmolC m^−3^ d^−1^. Unless otherwise stated, the supply of DOC is distributed evenly across all DOC compound units (see Supplement Fig. S11 for the influence of supply diversity on the DOC concentration, microbial biomass, and mean DOC age). For the simulation of mean DOC age (Fig. 5), we restricted the supply of DOC to 3% of compounds, to reflect a scenario where phytoplankton releases a limited subset of DOC compounds, which are molecularly diversified during subsequent microbial degradation^31,32,49^.

#### Uptake and release matrix (***U***, ***R***)

The default uptake matrix ***U*** = (***U***_*ij*_) is a binary matrix, where ***U***_*ij*_ = 1 encodes that the DOC compound unit *j* is taken up by microbial unit *i* according to the maximum uptake rate; ***U***_*ij*_ = 0 means that the DOC compound unit *j* is not taken up by microbial unit *i*. The matrix is randomly constructed in each model simulation with the following restrictions: i) all microbial units can take up the same number of substrates *n*_*U*_, i.e. the sum over the rows is identical, ii) all DOC compound units are associated with a decomposer, i.e. each column has at least one entry ***U***_*ij*_ = 1, and iii) there is no DOC compound unit which is consumed by all microbial units, i.e. each column has at least one entry ***U***_*ij*_ = 0. The sum over the columns, is on average (*m* × *n*_*U*_)/*n* = 1.05, however it differs slightly due to the random variations among the columns (standard variation of 0.23), thus each DOC compound unit has a slightly different total uptake rate.

The default release matrix ***R*** = (***R***_*ij*_) defines the partitioning of carbon among the release products, where ***R***_*ij*_ = 0.3 means that the microbial unit *i* releases 30% of released carbon in the form of compound unit *j*. A fixed number of release products (*n*_*R*_) per microbial unit is distributed randomly across the compound units. However, it is ensured that microbes do not release compound units they take up (i.e. ***R***_*ij*_ = 0, if ***U***_*ij*_ = 1). The row sums of the release matrix are equal to one, to preserve the mass balance of carbon. The sum over the columns differs due to random variations, thus each DOC compound unit has a slightly different total release rate (on average m/n = 0.35, with a standard deviation of 0.09).

The network of uptake and release abilities is constructed randomly. However, we show that the stochastic influence of the network on the long-term DOC concentration is low compared to the influence of the model parameters (see light green area in Fig. 3, indicating the variation between 50 randomly constructed networks). Similarly, Coles and colleagues^55^ found indications that biogeochemical gradients of DOC are determined by the total available pool of metabolic functions, rather than by the distribution of functions among organisms.

#### Energy turnover of microbes

To test the implications of the model parameterization on the assumed energy turnover of microbes, we calculated the energy gain from the complete oxidation of the DOC constituents for the individual microbial cells. The energy turnover of microbes is estimated assuming that the respiration in equilibrium matches the supply of DOC. According to LaRowe *et al*.^56^, the standard molal Gibbs energy in the model is 0.0054 kJ/m^3^/d, assuming the default supply rate (*s* = 8 × 10^−2^ mmolC/m^3^/d) and a mean nominal oxidation state of carbon of −0.2685 (based on 164 Atlantic DOM samples presented in Mentges *et al*.^57^). For a mean cell number in the bathypelagic of 6 × 10^10^ (0.12 mmolC/m^3^ ^35^ at 25 fgC/cell^58^), the energy turned over is 3.31 × 10^−11^ kJ/cell/year. This is orders of magnitude higher than the basal power requirements of microheterotrophs, estimated for oxic and anoxic sediments (3.31 × 10^−14^ kJ/cell/year and 10^−15^ to 10^−14^ kJ/cell/year^59^). Thus, in principle, microbial life in the deep sea can be sustained from the oxidation of DOC in a situation of dynamic steady-state^7^.

#### Simulating differential equations

The differential equations were solved numerically in MATLAB (Version 2017b, The MathWorks, Inc., Natick, Massachusetts, United States), using the non-stiff differential equation solver ode45, with the following options: maximum time step was 50 d, all state variables were set to be non-negative, and the relative error tolerance was set to 10^−6^. To prevent microbial biomass from dropping to the smallest positive normalized floating-point number in MATLAB (1 × 10^−308^) if no DOC is supplied, we added a constant influx of microbes at rate of 1 × 10^−300^ mmolC/m^3^/d. This corresponds to an inflow of roughly one microbial cell per day to a volume of 1 × 10^288^ m^3^ (carbon content of a single microbial cell ~2 × 10^−12^ mmolC^58^). As the volume of the ocean is about 1 × 10^18^ m^3^, this inflow of carbon is considered negligible relative to the state variables.

### Simulations

All simulations were initiated with a total biomass of 1 mmolC/m^3^ for the microbial community, representative for values typically observed in the surface ocean^35^. At 25 fgC/cell^58^, 1 mmolC/m^3^ of microbial biomass in the model translates to ~5 × 10^11^ cells.

#### Formation of a recalcitrant DOC pool

The amount of (apparently) recalcitrant DOC was determined for three different supply-scenarios (Fig. 2): high supply rates of DOC (default eutrophic supply *s* = 8 × 10^−2^ mmolC/m^3^/d), low supply rates of DOC (*s* = 8 × 10^−3^ mmolC/m^3^/d), and no supply of DOC (*s* = 0). The simulation was initiated with equal amounts of all DOC compound units and microbial units (in total 80 mmolC/m^3^ DOC, representing high surface-ocean values^34^). The flux to the inorganic carbon pool in the high, low, and no supply scenarios was ~1, 3, and 30 mmolC/m^3^ per year on average.

#### Size of the DOC reservoir: A sensitivity analysis

The steady-state DOC concentration was determined over a range of parameter values, based on 50 independent runs per parameter value (Fig. 3). Each parameter was varied individually, while the other parameters were kept at their default value. The parameters were varied between 50–150% of their default value. Note that the number of microbial units *m* could not be decreased by 50% without violating our basic assumption that every compound unit should be associated with a consuming microbial unit: at a default of 3 substrates per microbial unit and 100 compound units, at least 34 microbial units are required to maintain this basic assumption. Similarly, the number of compound units *n* could not be increased by 50%, and the number of substrates per microbe *n*_*U*_ could not be decreased by 50%.

#### Millennial scale stability of DOC

The age of DOC compound units was simulated in a Lagrangian water parcel moving along the oceanic circulation (Fig. 4, for derivation of age see supplement). The water parcel was assumed to stay in near-surface layers for one year, subsequently sink to deeper waters, remain in the deep sea for 899 years (i.e. average circulation age of water in the Pacific^60^), and finally rise back to the surface. The radiocarbon age is derived based on equations 4 through 12 in the supplement. The supply rate of DOC was variable over time to reflect the respective environmental conditions: high supply rates near the surface (default supply rate) and low supply rates of DOC in the deep sea from sinking particulate organic matter (one permil of the surface supply rate, as the global production of high molecular weight DOM in the deep ocean is estimated at 0.014 PgC/y^5^), which is ≈ 10^−3^ × 25 PgC/y surface labile DOC production^4^). The supply was linearly adapted to the new conditions after sinking/rising of the water parcel over a transition period of 10 days. To reflect the effect of an algal bloom, the supply of DOC was selectively distributed among the compounds, i.e. the total supply rate was split among a small, fixed subset of compound units. Note that we exclusively varied the DOC supply rate, whereas supply diversity was fixed throughout the simulation. The supply diversity was assumed at 3%, (see Supplement Fig. S11C for a sensitivity analysis of mean DOC age to this parameter). This setup reflects the observation that phytoplankton preferentially releases a limited subset of DOM compounds, which are molecularly diversified during subsequent microbial degradation^31,32,49^. To quantify the degree to which a DOC compound unit is supplied versus microbially produced, we derived a measure of the “distance to supply” for each compound unit (Fig. 5D,E, note that the term distance is not used in sense used in the context of networks, i.e. number of edges between two points, but rather depicts a proportion). It was calculated based on the total fraction of carbon released by consumers of supplied compounds. This measure was inverted and rescaled, such that 0 indicates that a compound is directly supplied e.g. from algae, whereas 1 indicates that the compound is exclusively microbially produced.

## Supplementary information

Supplementary Information
