# Supplementary material for: Long-term stability of marine dissolved organic carbon emerges from a neutral network of compounds and microbes
Source: Sci Rep. 2019 Nov 28;9:17780. doi: 10.1038/s41598-019-54290-z (PMC6883037; doi:10.1038/s41598-019-54290-z)
Supplement: Supplementary file 1 — Supplementary Information [file 41598_2019_54290_MOESM1_ESM.pdf]

Supplementary Materials

Equation describing the inorganic carbon pool

$$\dot{I} = (1-\beta)(1-\eta)\sum_{i=1}^{m}\sum_{j=1}^{n} \mathrm{U}_{ij}\frac{\rho D_j}{D_j+\kappa}B_i \tag{4}$$

Equations describing the DOC age

Assuming that consumed compounds' age impacts that of the heterotrophic biomass (*1*), we modelled the average age since fixation by primary producers for each DOM compound A_j^D and each microbial unit A_i^B as

$$\dot{A}_j^D = 1 - f_j^{supply}\left(A_j^D - a_j^{supply}\right) - f_j^{release}\left(A_j^D - a_j^{release}\right) - f_j^{mortality}\left(A_j^D - a_j^{mortality}\right), \tag{5}$$

$$\dot{A}_i^B = 1 - f_i^{uptake}\left(A_i^B - a_i^{uptake}\right). \tag{6}$$

Aging is influenced by the supply of compounds, by uptake and release of compounds, as well as mortality of microbial units, such that aging can be accelerated or slowed down. The degree to which a process impacts aging depends on its relative significance, which is given by the factors f_j^{supply}, f_i^{uptake}, $f_j^{release}$, and $f_j^{mortality}$, respectively:

$$f_j^{supply} = \frac{s_j}{D_j}, \tag{7}$$

$$f_i^{uptake} = \eta\sum_{k=1}^{n}\mathrm{U}_{ik}\frac{\rho D_k}{D_k+\kappa}, \tag{8}$$

$$f_j^{release} = \frac{\beta(1-\eta)}{D_j}\sum_{i=1}^{m}\mathrm{R}_{ij}\sum_{k=1}^{n}\mathrm{U}_{ik}\frac{\rho D_k}{D_k+\kappa}B_i, \tag{9}$$

$$f_j^{mortality} = \frac{1}{D_j}\sum_{i=1}^{m}\mathrm{R}_{ij}\mu B_i. \tag{10}$$

Whether a process accelerates or slows down aging (and also how much) depends on its associated average age a_j^{supply}, a_i^{uptake}, $a_j^{release}$, $a_j^{mortality}$, respectively. An average age exceeding that of the affected DOC compound A_j^D or microbial unit A_i^B speeds up aging, while lower ages slow down aging (or even lead to rejuvenation).

Assuming that supplied, i.e. newly produced DOC, has a modern radiocarbon age, we set its age $a_j^{supply} = 0$ years. Remaining ages, the average age a_i^{uptake} of compounds consumed by microbial unit *i*, the average age $a_j^{release}$ of DOC compound *j* when released after microbial transformation, and $a_j^{mortality}$, the average age of DOC compound *j* released by microbial lysis, must be computed dynamically:

$$a_i^{uptake} = \left(\sum_{k=1}^{n}\mathrm{U}_{ik}\frac{\rho D_k}{D_k+\kappa}\right)^{-1}\sum_{j=1}^{n}\mathrm{U}_{ij}\frac{\rho D_j}{D_j+\kappa}A_j, \tag{11}$$

$$a_j^{release} = \left(\sum_{i=1}^{m}\mathrm{R}_{ij}\right)^{-1}\sum_{i=1}^{m}\mathrm{R}_{ij}a_i^{uptake}, \tag{12}$$

$$a_j^{mortality} = \left(\sum_{i=1}^{m}\mathrm{R}_{ij}\right)^{-1}\sum_{i=1}^{m}\mathrm{R}_{ij}A_i^B. \tag{13}$$

Derivation of equilibrium value *D**

The equilibrium value is derived for a simplified version of the network model. The simplified version describes [1] the total microbial biomass concentration *B* as the sum of the concentrations of individual microbial units B_i, and [2] the total DOC concentration *D* as the sum of the concentrations of individual DOC compound units D_j. We define [3] the total supply of DOC compounds *s* as the sum of the supply of individual DOC compound units s_j, [4] the average uptake *u* as the average entry of the uptake matrix **U**, and [5] the average release *r* as the average entry of the release matrix **R**. Assuming that the uptake and release matrices are constructed as specified in the Methods section, the average uptake and release can be substituted by a ratio of model parameters $u = \frac{n_U}{n}$ and $r = \frac{1}{n}$. The Michaelis-Menten kinetic is approximated based on a Taylor polynomial of degree 1 at $D_j = 0$, i.e. $\frac{\rho D_j}{D_j+\kappa} \approx \frac{\rho D_j}{\kappa}$, assuming that the concentration of individual DOC compounds (not the bulk DOC) are very small in the ocean. Combining those simplifications to approximate total microbial biomass *B* and total DOC concentration *D,* the full model reduces as follows:

$$\dot{B} = \sum_{i=1}^{m} \eta \sum_{k=1}^{n} u \frac{\rho D_k}{D_k + \kappa} B_i - \sum_{i=1}^{m} \mu B_i$$
$$\approx \eta u \sum_{k=1}^{n} \frac{\rho D_k}{D_k + \kappa} B - \mu B$$
$$\approx \eta u \sum_{k=1}^{n} \frac{\rho D_k}{\kappa} B - \mu B$$
$$= \eta u \frac{\rho}{\kappa} DB - \mu B$$
$$= \eta \frac{n_U}{n} \frac{\rho}{\kappa} DB - \mu B$$

$$\dot{D} = \sum_{j=1}^{n} \left(- \sum_{i=1}^{m} u \frac{\rho D_j}{D_j + \kappa} B_i + \sum_{i=1}^{m} r \mu B_i + \beta(1-\eta) \sum_{i=1}^{m} r \sum_{k=1}^{n} u \frac{\rho D_k}{D_k + \kappa} B_i + s_j \right)$$
$$\approx - \sum_{j=1}^{n} \sum_{i=1}^{m} u \frac{\rho}{\kappa} D_j B_i + \sum_{j=1}^{n} \sum_{i=1}^{m} r \mu B_i + \beta(1-\eta) \sum_{j=1}^{n} \sum_{i=1}^{m} r \sum_{k=1}^{n} u \frac{\rho}{\kappa} D_k B_i + \sum_{j=1}^{n} s_j$$
$$= -u \frac{\rho}{\kappa} DB + \sum_{j=1}^{n} r \mu B + \beta(1-\eta) \sum_{j=1}^{n} r u \frac{\rho}{\kappa} DB + s$$
$$= - \frac{n_U}{n} \frac{\rho}{\kappa} DB + \mu B + \beta(1-\eta) \frac{n_U}{n} \frac{\rho}{\kappa} DB + s$$

Thus, in the case of average DOC decomposition, the differential equations simplify to the following:

$$\dot{B} = \eta \frac{n_U}{n} \frac{\rho}{\kappa} DB - \mu B$$

[14]

$$\dot{D} = -\frac{n_U}{n}\frac{\rho}{\kappa}DB + \mu B + \beta(1-\eta)\frac{n_U}{n}\frac{\rho}{\kappa}DB + s\ .$$

[15]

Assuming that the concentration of microbial biomass and DOC are constant in equilibrium, $\dot{B} = 0, \dot{D} = 0$, the long-term average concentration of DOC can be derived, (corresponding to equation 1 from the main text): $D^{*} = \frac{\mu\,\kappa\,n}{\eta\,\rho\,n_U}$.

Supplementary Figures

Concentration distribution of DOC compounds (in addition to Fig. 2)

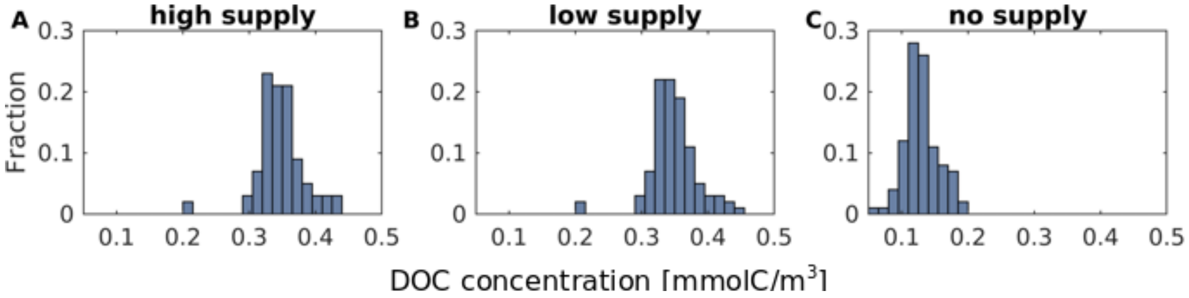

Fig. S1. Concentration distribution of individual DOC compound units. The concentration distribution of DOC compounds after 100 simulation years for (**A**) high, (**B**) low, and (**C**) no supply of DOC. The concentration distribution of modelled compounds resembles the gaussian-shaped distribution of DOM compounds typically found using ultra-high resolution mass spectrometry (e.g. (*2*, *3*)). The variability in the concentration of DOC compounds is caused by a slight variability in the total uptake and release rate of each compound (while the row sums of the release matrix are fixed to ensure equal number of released compounds per bacteria, the column sums of the release matrix vary). The higher the total uptake rate of a compound unit, the lower its long-term concentration (according to Figure 3A). The small differences in the concentration distribution in panels A and B arise due to the non-linear relationship between DOC concentrations and microbial growth.

Microbial biomass for varying model parameters (in addition to Fig. 3)

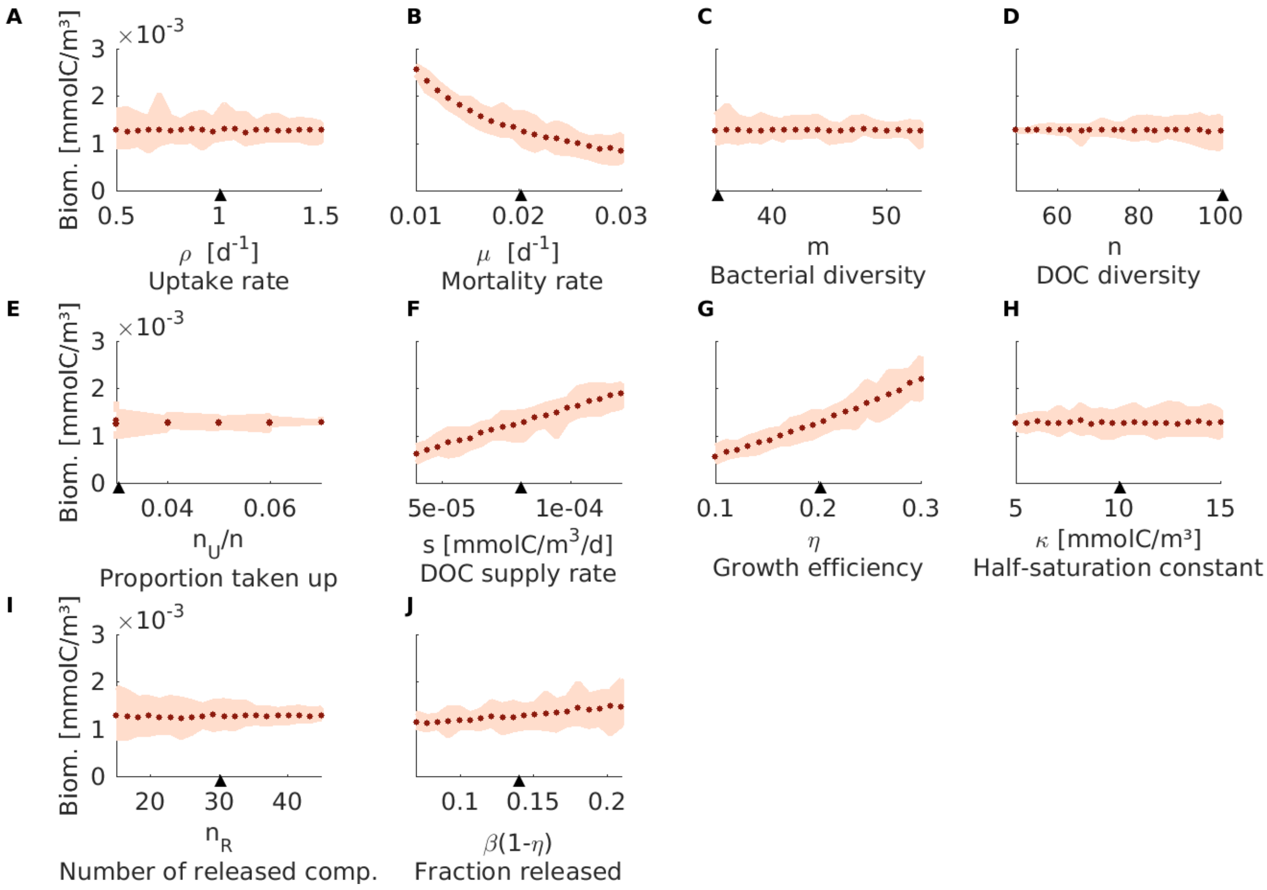

Fig. S2. Sensitivity of microbial biomass to parameter variations. The total concentration of microbial biomass after 20,000 simulation years, based on varying parameter values of: (**A**) the microbial uptake rate ρ, (**B**) the microbial mortality rate μ, (**C**) the number of microbial units *m*, (**D**) the number of DOC units *n*, (**E**) the proportion of total number of compounds which can be taken up by a single microbial unit n_U/n (note that here n_U is varied, while *n* is fixed), (**F**) the total supply rate of DOC *s*, (**G**) the microbial growth efficiency η, (**H**) the half-saturation constant of microbial DOC uptake κ, (**I**) the number of DOC compound units released per microbial unit n_R, and (**J**) the fraction of taken up carbon released as transformed compounds to the DOC pool β(1-η). Each simulation was repeated 50 times, the red dots indicate the mean, the light orange area represents the minimum and maximum concentrations from the 50 runs. The black triangle indicates the default value of the parameter.

DOC age for varying model parameters (in addition to Fig. 3)

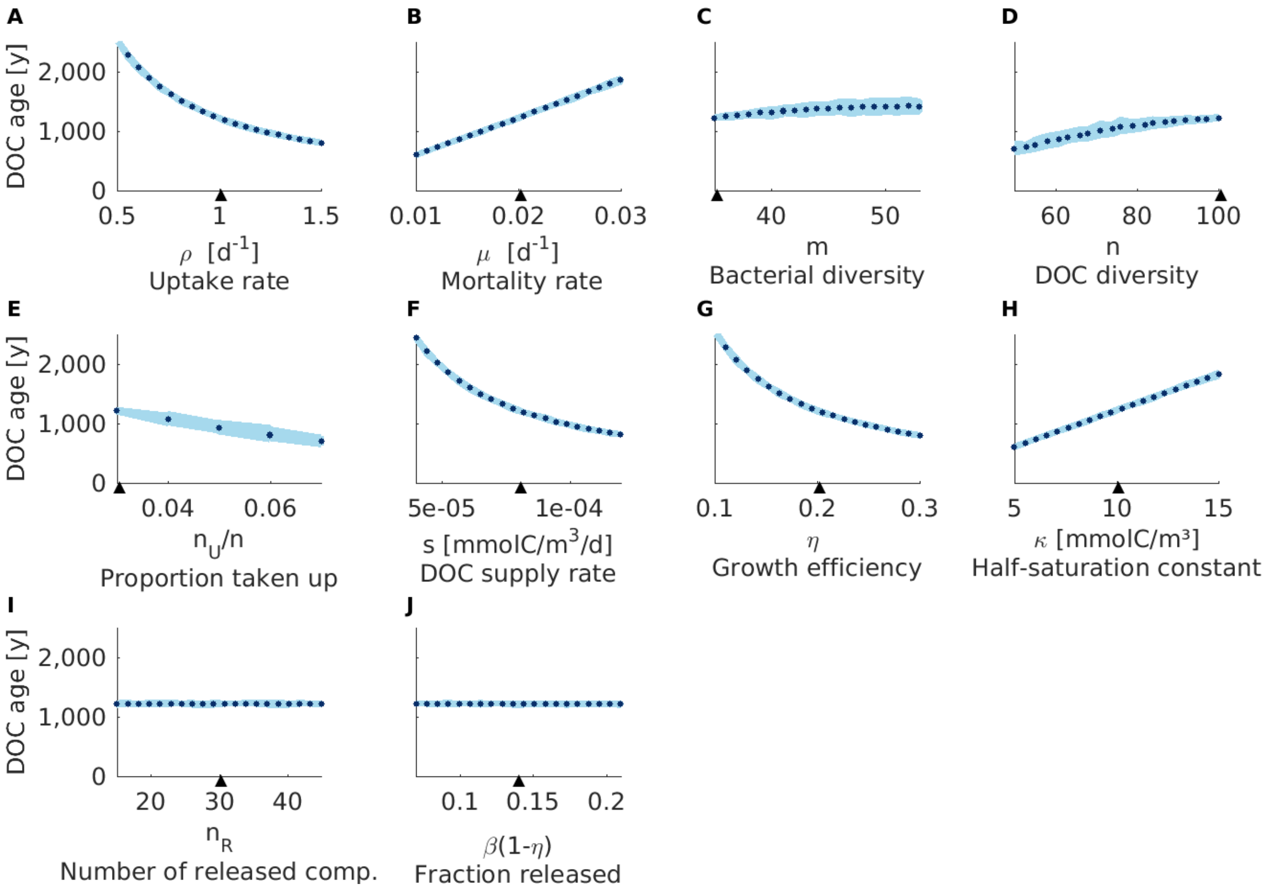

Fig. S3. Sensitivity of DOC age to parameter variations. The mean age of DOC after 20,000 simulation years, based on varying parameter values of: (**A**) the microbial uptake rate ρ, (**B**) the microbial mortality rate μ, (**C**) the number of microbial units *m*, (**D**) the number of DOC units *n*, (**E**) the proportion of total number of compounds which can be taken up by a single microbial unit n_U/n (note that here n_U is varied, while *n* is fixed), (**F**) the total supply rate of DOC *s*, (**G**) the microbial growth efficiency η, (**H**) the half-saturation constant of microbial DOC uptake κ, (**I**) the number of DOC compound units released per microbial unit n_R, and (**J**) the fraction of taken up carbon released as transformed compounds to the DOC pool β(1-η). Each simulation was repeated 50 times, the blue dots indicate the mean, the light blue area represents the minimum and maximum concentrations from the 50 runs. The black triangle indicates the default value of the parameter. Note that the average age of DOC is lower in this figure compared to Fig. 5, as in this exercise there is no Lagrangian circulation and all compounds are supplied at the same rate (uniform supply distribution according to the default parameterization of the model, see Fig. S11 for details on the relationship between age and supply diversity).

Size of the DOC reservoir: Remaining parameters (in addition to Fig. 3)

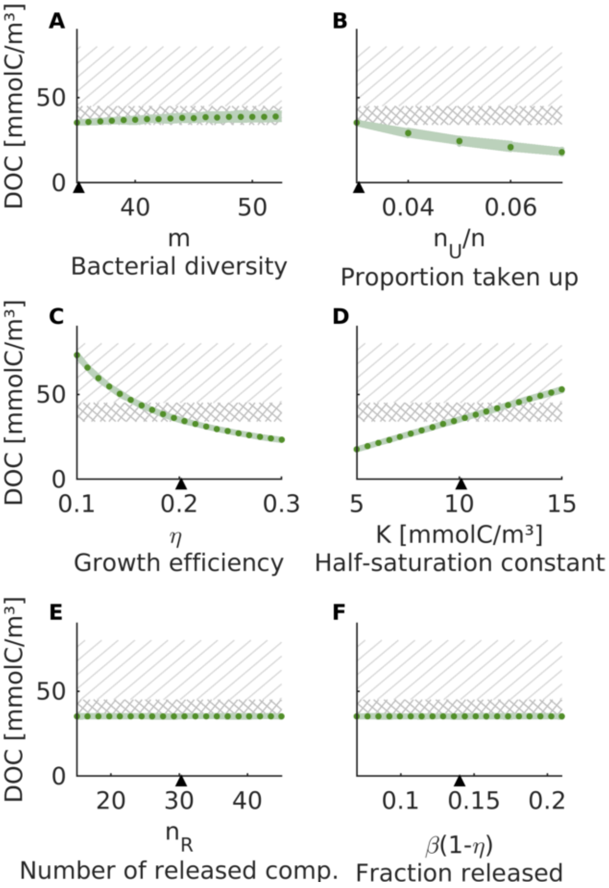

Fig. S4. Variation of model parameters. The total concentration of DOC after 20,000 simulation years, based on varying parameter values: (**A**) the number of microbial units *m*, (**B**) the proportion of total number of compounds which can be taken up by a single microbial unit n_U/n (note that here n_U is varied, while *n* is fixed), (**C**) the microbial growth efficiency η, (**D**) the half-saturation constant of microbial DOC uptake κ, (**E**) the number of DOC compound units released per microbial unit n_R, and (**F**) the fraction of taken up carbon released as transformed compounds to the DOC pool β(1-η). Each simulation was repeated 50 times, the green dots indicate the mean, the light green area represents the minimum and maximum concentrations from the 50 runs. The black triangle indicates the default value of the parameter. The hatched region indicates the range of DOC concentrations typically observed in the surface ocean, the cross-hatched region indicates typical deep sea DOC values (*8*, *9*).

The steady-state DOC concentration is not significantly influenced by the number of microbial units *m* (Fig. S4A). Intuitively, it might be expected that adding more microbial units introduces new degradation abilities to the microbial community. However, in the model, each DOC compound unit is degraded by at least one microbial unit. Thus, the microbial community is always able to degrade any model-DOC-compound, irrespective of the number of microbial units. Through the addition of microbial units, the number of microbial units consuming a specific DOC compound might increase. However, the concentration of the individual microbial units is reduced as the total amount of available substrate remains unchanged. Therefore, increasing the number of microbial units does not increase the overall feeding pressure of microbes on DOC, but simply splits the same biomass among more microbial units.

The number of compound units released per microbial unit n_R also shows no effect on the steady-state DOC concentration (Fig. S4E). This is because n_R only affects the diversification speed of carbon, but not the amount of carbon available.
Similarly, the fraction that is recycled to DOC $\beta(1-\eta)$ does not affect the steady-state DOC concentration (Fig. S4F), because an increase of $\beta(1-\eta)$, only reduces the fraction that is respired to inorganic carbon $(1-\beta)(1-\eta)$, whereas the fraction that is fixed to microbial biomass η remains the same.
The turn-over of DOC by microbes is fuelled by an increase in the proportion of compounds taken up per microbial unit n_U/n and in the microbial growth efficiency. Therefore, these parameters constrain the size of the DOC reservoir (Fig. S4B,C). In contrast, the half-saturation constant κ limits the microbial DOC uptake. Accordingly, the size of the DOC pool increases with increasing half-saturation constant (Fig. S4D).

Impact of the least constrained parameters on the DOC concentration

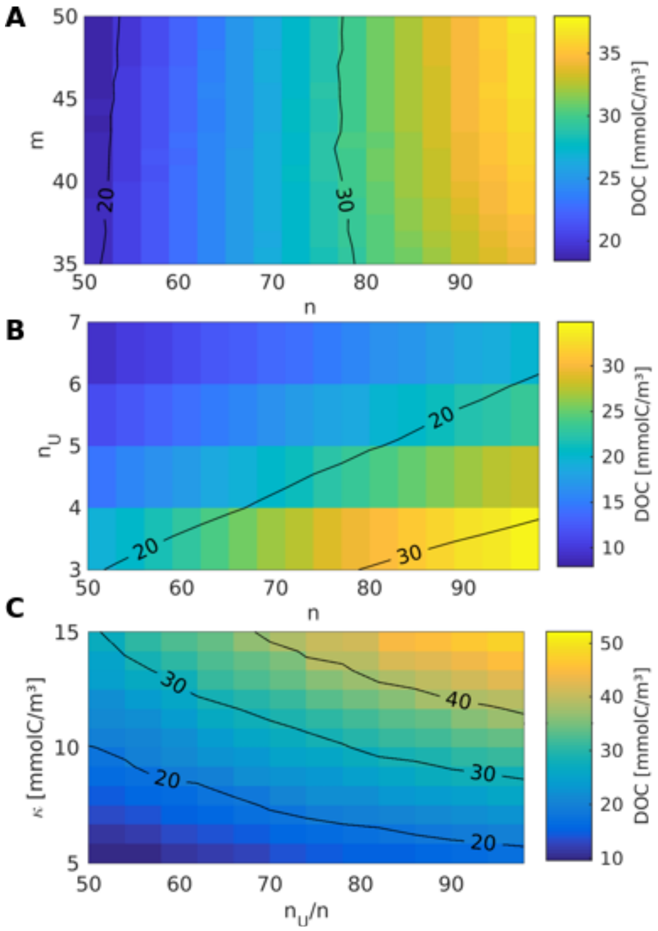

Fig. S5. Sensitivity of DOC concentration to variations in the least constrained parameters. The steady-state DOC was determined for a range of number of microbial units *m*, number of DOC compounds *n*, number of substrates per microbial unit n_U, and half-saturation constants κ, based on the average of 100 runs per combination. The steady-state DOC concentration depends strongly on the number of compound units taken up by a microbial unit n_U, the total number of compound units *n,* and the half-saturation constant of microbial uptake κ. (**A**) The steady-state DOC concentration depends strongly on the number of compound units *n,* whereas the number of microbial units *m* is not influencing the steady-state DOC (see also Fig. S4A and respective discussion). (**B**) Further, the steady-state DOC concentration is influenced by the ratio n_U/n, i.e. the percentage of DOC compound units that can be consumed by a single microbial unit, and (**C**) the half-saturation constant κ.

Microbial biomass in the regime shift simulation (in addition to Fig. 5)

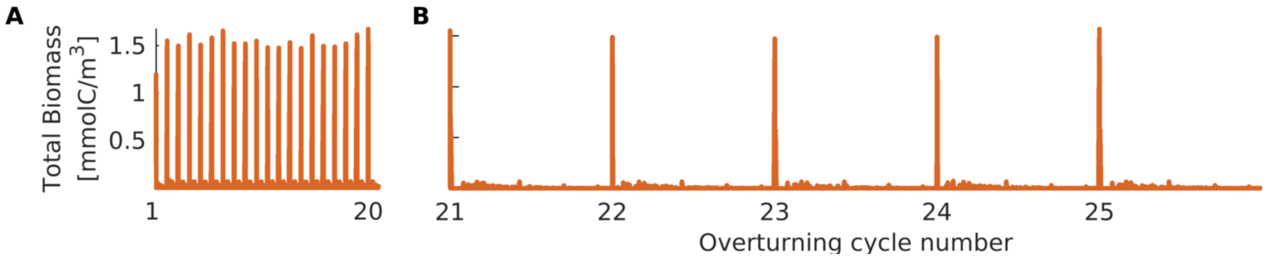

Fig. S6. Microbial biomass in the age experiment. The transport of a water parcel by ocean circulation is simulated by varying DOC supply in 25 overturning cycles: at the beginning of each cycle, one year of surface conditions is simulated in the form of high supply of DOC, whereas for the following 899 years low supply rates of DOC simulate deep-sea conditions (see Fig. 5). The variations in total supply rate influence the total microbial biomass concentration. The left panel (**A**) shows the first 20 overturning cycles, during which the system approaches a dynamic equilibrium. The right panel (**B**) shows the last five overturning in detail. See main text Fig. 5 for the respective time series of DOC concentration and DOC age. Note that our model does not include particle associated microbes, and as such our values for total biomass are lower than those reported in the literature.

Consistency of the model results with an incubation experiment (*5*)

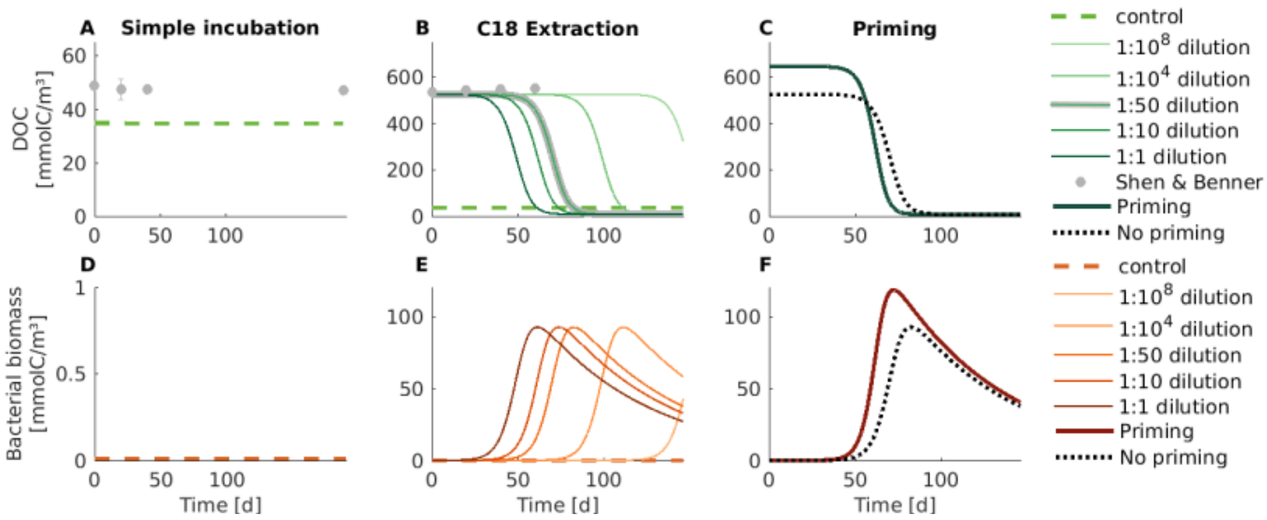

Fig. S7. Persistence of modelled DOC, inspired by an incubation experiment of C-18 extracted DOC by Shen and Benner (*5*)**.** (**A**, **D**) The virtual deep-sea sample of DOC and a microbial community is in steady state. (**B**, **E**) After addition of DOC and dilution of microbes, the microbial community slowly grows and reduces the DOC concentration to a lower level. The coloured lines indicate different dilution factors of the microbial community. The dashed line indicates a control, where DOC was not enhanced and microbes were not diluted. The grey dots indicate the experimental results from Shen and Benner where the microbial community was diluted 1:50 (bold line) (*5*). (**C**, **F**) Priming with 120 mmolC/m^3 glucose results in stronger growth of the microbial community, leading to faster consumption of DOC in the model similar to the observation in Shen and Benner (*5*).

The persistence of DOC was simulated in a virtual incubation inspired by an experiment by Shen and Benner (*5*). The authors incubated C-18 solid phase extracted DOC from the Atlantic Ocean with a 1:50 diluted microbial community. We used the virtual deep-sea DOC and microbial community which served as a basis for Fig. S8 (run with a supply rate of $s = 8{\times}10^{-4}$ mmolC/m^3/d, see Methods section for more details).
This sample was enhanced with DOC to a total concentration of 525 mmolC/m^3, corresponding to the 1,200m-sample from Shen and Benner. The microbial community was diluted in a ratio between 1:1 and 1:10^8 and the model was run without supply of DOC for 146 simulation days. To simulate priming, the 1:50 diluted sample received an addition of 120 mmolC/m^3 glucose.

In the virtual deep-sea sample, a steady-state of DOC and microbial biomass emerged, i.e. no significant microbial consumption took place (Fig. S7A,D). After addition of DOC and dilution of microbes, the microbial community slowly increased in biomass (Fig. S7E). During the initial phase of growth, DOC concentrations remained roughly constant (Fig. S7D). The duration of this growing phase increases with the dilution factor. For a dilution of 1:50, corresponding to the Shen and Benner experiment, DOC declines from day ~60 onward. However, for a 1:10^8 dilution, the bloom phase of the microbes is not observed within the duration of the virtual experiment. We hypothesize that the apparent recalcitrance of C-18 extracted DOC in the incubation by Shen and Benner can be

explained by the dilution of microbes, i.e. very low cell numbers. Accordingly, the higher DOC removal of a coastal community reported by Shen and Benner can be explained by higher cell numbers.

A priming effect can be observed in the C-18 and the priming experiment (Fig. S7B,C) due to the strong increase in DOC concentration: the microbial community reduces the DOC concentration to 20 % the level of the simple incubation control (indicated by the dashed line, Fig. S7B). Priming of DOC by a large addition of glucose leads to increased growth of microbes (Fig. S7C,F). During the first 60 days, utilization of glucose is observed while C-18 extracted DOC is not consumed. This time period of DOC persistence could be longer in real laboratory experiments than in the model. Our model was designed to study whether DOC can persist at realistic concentrations in the deep ocean if all compounds were labile. To conservatively test this, we assume high uptake rates of DOC and an "omnipotent" microbial community (i.e. which can degrade any DOC substrate). If some functional groups of microbes are absent in the initial sample of Shen and Benner, a small fraction of compounds is consumed with lower uptake rates, or physiological stress occurs - all of which is not part of the simulation setup - the time span of DOC persistence in the experiment would increase relative to that of the simulation. This limitation is relevant on short timescales of up to a year but become increasingly negligible when time scales increase.

Consistency of the model results with an experiment by Arrieta et al. (*4*)

The concentration-dependence of microbial uptake was tested based on an experimental set-up by Arrieta et al. (*4*). The authors incubated deep-sea microbial communities with four concentration levels of DOC. We generated deep-sea controls based on a simulation run with very low supply until steady state (supply rate of $s = 8\times10^{-4}$ mmolC/m^3/d). The supply rate was adjusted to result in total microbial biomass and DOC concentrations in the same order of magnitude as the controls from Arrieta et al. (0.01 mmolC/m^3 microbial biomass in our model versus ~0.0007-0.01 mmolC/m^3 in the experiment, and ~35 mmolC/m^3 versus ~37-50 mmolC/m^3 DOC, respectively). The deep-sea microbial community was then virtually incubated for three years, starting either with ambient (control), 2-fold, 5-fold, or 10-fold increased DOC concentrations, without further supply of DOC.

Apparently recalcitrant DOC from a previous simulation is taken up by our virtual microbial community if concentrated, according to our assumption of neutral reactivity, and resulting in increased microbial biomass (Fig. S8). During the first 15 days of simulation (run without supply), the maximum microbial biomass in our 10-fold concentration simulation increases by a factor of 5.6 in comparison to the control (see magnification of Fig. S8B in the grey box), whereas the DOC concentration remains relatively constant (Fig. S8A, utilization of ~1 ‰ of DOC for all treatments until day 15). During the following three simulated years, the DOC concentration is reduced, as the microbial community completes its bloom and shows an early stationary phase (the consumption of DOC after concentration has been attributed to the method of extraction (*5*), see Fig. S7 for a comparison of the model results to this study). The more concentrated the deep-sea DOC, the higher is its microbial utilization: the percentage of DOC taken up by microbes after three years increases from 0.02 % in the control to ~76, 95 and 98 % in the 2-fold, 5-fold and 10-fold experiment, respectively. The time scale of the bacterial bloom (and the main DOC decomposition) is determined by the growth rate, uptake kinetics, and by the initial microbial biomass. If the microbes are present in very low cell numbers, as in the deep ocean, more time is needed until the population reaches its peak. Also, the initial DOC concentration influences the time scale of the bloom, higher initial DOC concentrations facilitate the quick growth of microbes.

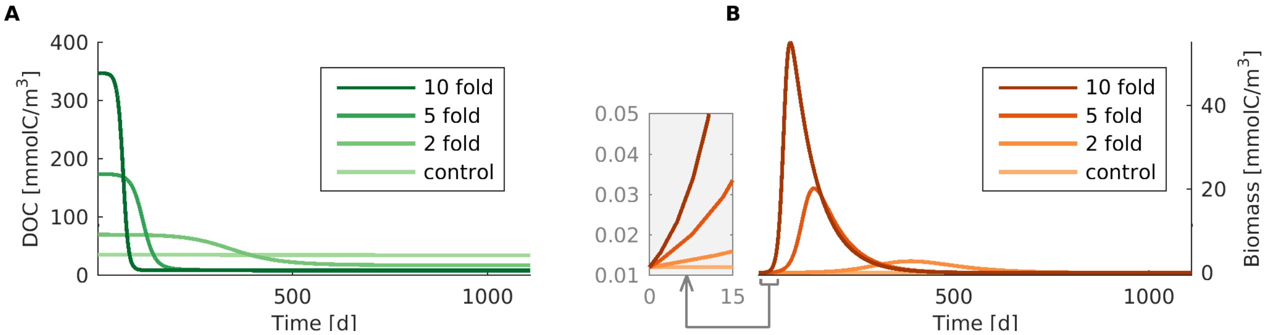

Fig. S8. Uptake of concentrated DOC. Simulated incubation experiments on deep-sea DOC, inspired by a study of Arrieta et al. (*4*). Four levels of initial DOC concentrations (10-fold, 5-fold, 2-fold, and control concentration) are shown with their resulting (**A**) total DOC concentration, and (**B**) total microbial biomass over time. The magnification of the microbial biomass time series shows the first 15 days of the incubation. Concentrated deep-sea DOC facilitates a microbial bloom, as indicated by increased microbial biomass.

This model outcome is consistent with results from an experiment by Arrieta et al. (*4*), where a deep-sea microbial community was incubated for 10-22 days with ambient, 2-fold, 5-fold, and 10-fold concentrated deep-sea DOC. The authors report microbial growth on the concentrated DOC.

Our model reproduces the partial utilization of apparently recalcitrant DOC after concentration (Fig. S8, (*4*)). The concentration of DOC causes an increase of the uptake rate according to Michaelis-Menten kinetics. As the uptake increases relative to the concentration-independent mortality rate, a net increase of microbial biomass occurs. The uptake of apparently recalcitrant DOC and the associated increase in biomass thus result from the implementation of basic microbiological principles in the model.

In the experiment by Arrieta et al. (*4*), microbial biomass increased by a factor of 3.6-11.7); the increase by a factor of 5.6 after 15 days in our model falls into this range. In a comment to the experiment, Jiao et al. (*6*) pointed out, that for dilution-limited growth, the utilization of DOC should increase with its concentration. In the incubations from Arrieta and colleagues, which were shorter than 22 days, this was not the case. The same applies to the first 15 days of our simulation, where DOC concentration is essentially constant, and in laboratory experiments such changes would be hardly detectable analytically. However, during the following three simulation years, the utilization rate increases strongly, similar to the predicted increase by Jiao et al. (*6*) (from ~5 % in the control to about 50, 80, and 90 %, in the model: ~0.02, 76, 95, and 98, respectively).

In a study by Sosa et al. (*7*), enrichment of bacterial isolates with high-molecular weight DOM increased bacterial cell numbers, but only a fraction of DOM was remineralized. The authors state that likely several metabolic strategies exist for the degradation of DOM and microbes degrade DOM collectively. Similarly, in our model, if a subset of the microbial community was isolated, more DOC would persist, because some pathways of DOC degradation in the uptake and release network would be lost.

Alternative model set-up: Intrinsic stability

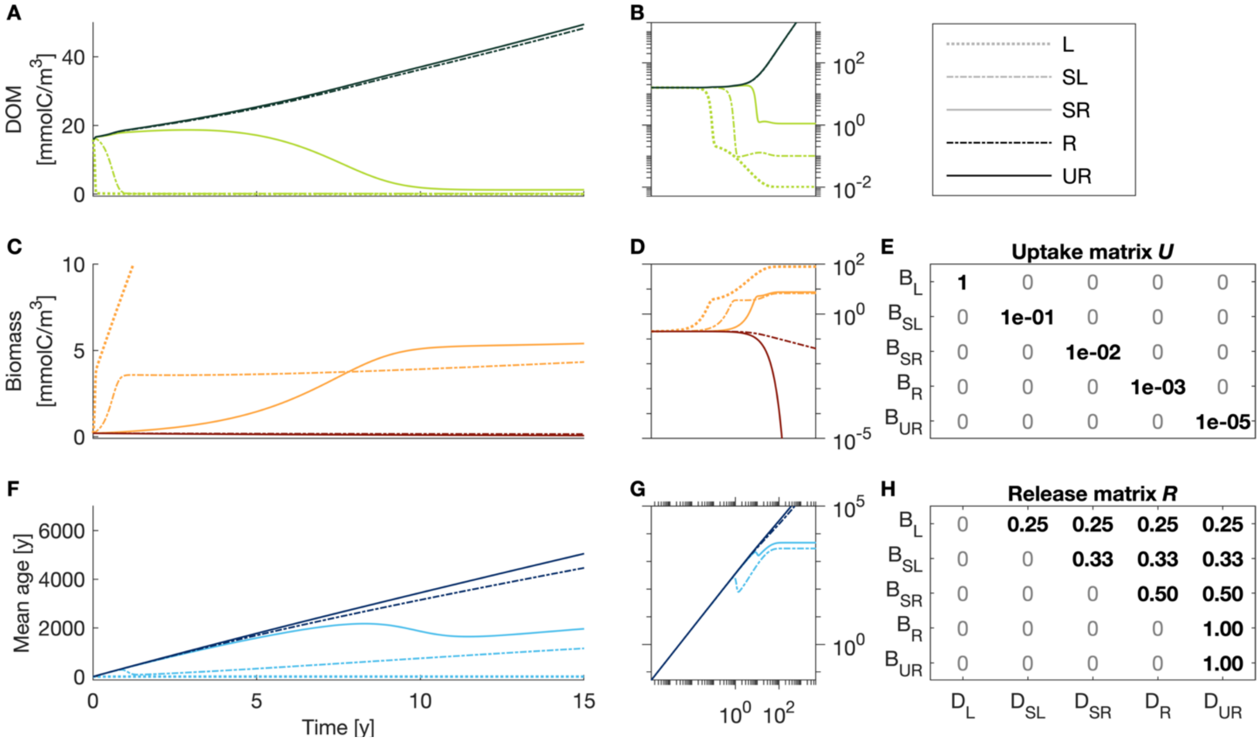

Fig. S9. Alternative model set-up, assuming the five traditional reactivity classes of DOC. (**A**) Total DOC concentration, (**C**) total microbial biomass, and (**F**) mean DOC age over 15 simulation years. (**B**, **D**, **G**) The same data are shown on a double-logarithmic scale over 500 simulation years. Five reactivity classes of DOC supply are shown: labile (L, light dotted line), semi-labile (SL, light dot-dashed line), semi-refractory (SR, light solid line), refractory (R, dark dot-dashed line), and ultra-refractory DOC (UR, dark solid line), microbial consumers and DOC age classes follow the same line coding. The network of uptake and release preferences is visualized in the form of (**E**) the uptake matrix and (**H**) the release matrix.

In an alternative model scenario, we implemented the five traditional reactivity classes of DOC according to Hansell (*10*): labile (L), semi-labile (SL), semi-refractory (SR), refractory (R), and ultra-refractory (UR) DOC (Fig. S9). Each reactivity class is associated with a group of microbes who can take it up. Labile DOC is taken up proportional to the default maximum uptake rate $\mu_L = \mu$. To account for lower reactivity, the maximum uptake rate is reduced in the other reactivity classes: $\mu_{SL} = 0.1\times\mu$, $\mu_{SR} = 0.01\times\mu$, $\mu_R = 0.001\times\mu$, and $\mu_{UR} = 1\times10^{-5}\times\mu$. Orders of magnitude of reactivity (i.e. maximum uptake rates) are based on removal rates compiled in Dittmar and Stubbins (*11*).
To include the variability in uptake rates among the microbial units in our model, we adapted the entries of the consumption matrix, which serve as factors for the maximum uptake rate (Fig. S9E). The microbial units release all less reactive DOC classes in equal amounts (Fig. S9H). The decomposers of URDOC release URDOC again (note that this violates our original restriction that microbial units do not release compounds they can take up). The supply of DOC (with default eutrophic supply rate *s*) is restricted to LDOC. This scenario reflects the view of Jiao and colleagues (*12*): algae produce labile DOC, whereas microbes produce recalcitrant DOC.

The run was initiated with a total DOC concentration of 80 $mmolC/m^3$ and a total microbial biomass of 1 $mmolC/m^3$, run over 1,000 simulation years. The microbial mortality rate was decreased by a factor of 1,000, as otherwise only decomposers of LDOC were able to maintain a stable population, and all other DOC reactivity classes accumulated. However, this strong reduction of the mortality rate increases microbial biomass levels to >100 $mmolC/m^3$.

The decomposers of DOC can be split in two groups, based on their long-term behaviour. The first group of decomposers, consuming LDOC, SLDOC, and SRDOC, reaches a steady-state concentration: their growth on supplied and/or microbially produced DOC balances mortality (shown in light orange in Fig. S9C, D). Accordingly, their consumed DOC fractions too reach a steady-state concentration: consumption, release, and supply balance (shown in light green in Fig. S9A, B). Decomposers of LDOC bloom first as they have the highest uptake rates. They also maintain the highest level of steady-state biomass due to the supply of labile DOC. The second group of decomposers, consuming RDOC and URDOC, declines towards zero, as it receives too little input to maintain a higher biomass level. As a consequence, RDOC and URDOC accumulate until the end of the simulation (shown in dark green in Fig. S9A, B). In contrast to our default model (i.e. assuming all DOC compound units are labile), the total DOC concentration at the end of the simulation doubles, if the supply rate of labile DOC is doubled, due to the accumulation of URDOC.
Any number of intermediate reactivity classes could be implemented between LDOC and URDOC. They would follow the basic principles illustrated here. Even if the production matrix is altered drastically (e.g. each microbial unit produces the next-less-reactive DOC class, and decomposers of URDOC produce LDOC), the basic behaviour of this reactivity-class-model remains the same: DOC accumulates continuously and does not reach a steady-state. A steady-state DOC concentration is only reached during this 500-year simulation if, a) the total supply rate of DOC is reduced by a factor of a millionth to 8×10^{-8} $mmolC/m^3/d$, in addition to the reduction of mortality rate by a factor of 1,000. Then, the total microbial biomass at the end of the simulation is 1×10^{-4} $mmolC/m^3$, and the total DOC concentration is ~41 $mmolC/m^3$. Or, b) efficient abiotic removal mechanisms are assumed to balance the steady input of DOC. These potential mechanisms would need to remove 99.99990% of the supplied DOC in order to stabilize DOC concentrations on the 500-year simulation time-scale (corresponding to a decreased net supply rate of 8×10^{-8} $mmolC/m^3/d$). Such efficient removal mechanisms are still up for discussion (*10*).

Re-working of DOC in the network model

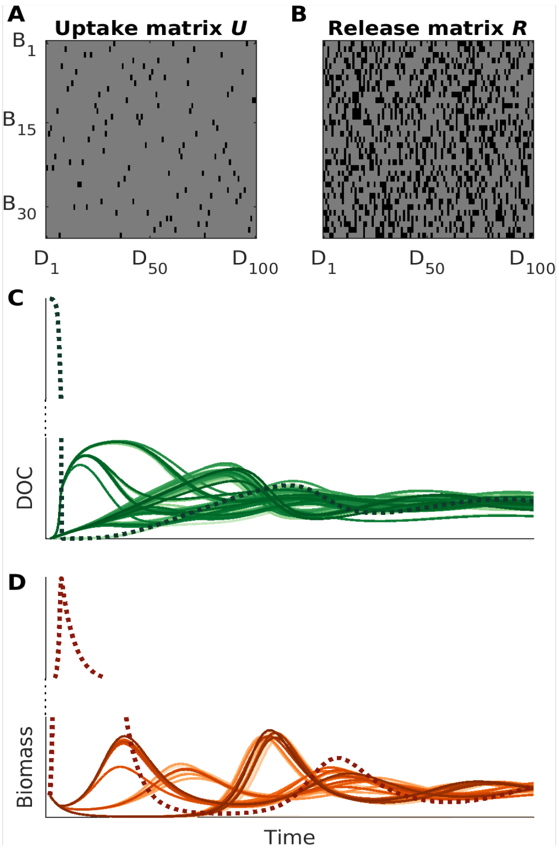

Fig. S10. Illustration of the re-working of DOC in the network model . The transformation network of DOC is defined by two matrices, (**A**) the uptake matrix **U**, and (**B**) the release matrix **R**, where grey blocks indicate entries of zero. The model predicts (**C**) the concentration of individual DOC compound units $D_1, \ldots, D_{100}$ and (**D**) the biomass of each microbial unit $B_1, \ldots, B_{35}$ over time. Note the break in the vertical axis in panels C-D. At the start of the simulation, a single DOC compound unit is provided in high concentrations as the initial substrate, indicated by the dotted green line. The dotted orange line indicates the microbial unit that is specialized on the degradation of the initial substrate.

To illustrate the basic model behaviour, we present a simulation initialized with a single substrate compound unit at a concentration of 80 mmolC/m^3 and equal amounts of each microbial unit of 1 mmolC/m^3 in total (Fig. S10). In this virtual incubation, there is no supply of DOC. The initial substrate is degraded by a microbial unit specialized on its decomposition (microbial unit number 22, see uptake matrix, Fig. S10A) and thus decreases in concentration (Fig. S10C). Accordingly, the microbial unit specialized on this substrate strongly increases in biomass (Fig. S10D). During its growth, the microbial unit releases DOC in the form of 30 other compound units (see release matrix, Fig. S10B), which enables other microbial units to grow and release other compounds. The single initial substrate is thus diversified by the collective microbial community. The principle is also illustrated in detail using a toy-model set-up with a small number of microbial and compound units (Fig. 7). Due to the respiration of organic carbon to inorganic carbon (time series not shown) the total concentration of DOC declines over time.

Sensitivity to supply diversity

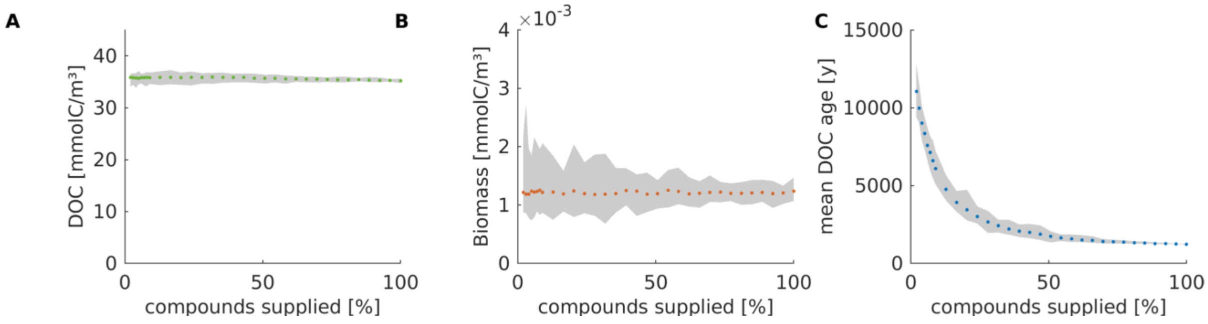

Fig. S11. Sensitivity of mean DOC age to the percentage of supplied compounds. The influence of supply diversity (i.e. percentage compounds supplied) on (**A**) DOC concentration, (**B**) microbial biomass, and (**C**) concentration-weighted mean DOC age is shown after 100 simulation years. The simulation was repeated for 100 times, the blue dots indicate the mean, the light grey area indicates the minimum and maximum concentrations from the 100 runs.

To determine how the mean DOC age depends on the number of compounds that are supplied via primary production, the total supply rate of DOC *s* was fixed at the default supply rate of 8×10^{-2} mmolC/m^3/d and the total supply was distributed evenly among the supplied compounds. The mean age of DOC was lowest (~1 year), when all DOC compound units were supplied (Fig. S11C). This corresponds to a scenario where phytoplankton releases all DOC compounds that are found in the ocean, including thermally altered and riverine compounds. The mean age of DOC increased when fewer compound units were supplied, reaching its maximum at the supply of a single DOC compound unit, representing a scenario where phytoplankton releases one percent of all DOC compounds found in the oceans. The supply diversity did not influence the average long-term DOC concentration and microbial biomass (Fig. S11A,B). Accordingly, the results presented in Fig. 2 and Fig. 3 are not sensitive to the choice of this parameter. The mean age shown in Fig. 5, in contrast, crucially depends on the diversity of compounds supplied. We chose a supply diversity of 3% for this simulation, to reflect a scenario where phytoplankton preferentially releases a limited subset of DOC compounds, which are molecularly diversified during subsequent microbial degradation (*13–15*). To generate realistic mean ages of DOC, the model thus relies on the assumption that microbes strongly diversify organic compounds.

Supplementary Tables

Table S1. List of symbols and abbreviations.

Abbreviation	Description
(1-η)(1-β)	Fraction of uptake respired to inorganic carbon
β(1-η)	Fraction of uptake released to DOC
η	Fraction of uptake converted to microbial biomass
μ	Microbial mortality rate
ρ	Maximum microbial uptake rate
κ	Half-saturation constant of microbial uptake
A_i^B	Age of carbon that is stored as biomass in microbial unit *i*
A_j^D	Age of carbon in DOC compound unit *j*
a_i^{uptake}	Age of carbon that is consumed by microbial unit *i*
$a_j^{mortality}$	Age of carbon from microbial mortality that is provided in the form of compound unit *j*
$a_j^{release}$	Age of carbon that is released by microbes in the form of compound unit *j*
a_j^{supply}	Age of carbon that is supplied in the form of compound unit *j*
B_i	Carbon concentration of microbial unit *i*
D_j	Carbon concentration of DOC compound unit *j*
DOC	Dissolved organic carbon
DOM	Dissolved organic matter
f_i^{uptake}	Fraction of total carbon in microbial unit *i* that is added via uptake
$f_j^{mortality}$	Fraction of total carbon in compound unit *j* that is added via microbial mortality
$f_j^{release}$	Fraction of total carbon in compound unit *j* that is added via microbial release
f_j^{supply}	Fraction of total carbon in compound unit *j* that is added via supply
I	Carbon concentration of the inorganic carbon pool
i	Index of a microbial unit ($i = 1, 2, \ldots, m$)
j	Index of a compound unit ($j = 1, 2, \ldots, n$)
m	Total number of microbial units
n	Total number of compound units
n_R	Number of compound units released by a single microbial unit
n_U	Number of compound units taken up by a single microbial unit
$\boldsymbol{R}=(\boldsymbol{R}_{ij})$	Release matrix, defines which DOC compound units are released by which microbial unit
$\boldsymbol{R}_{ij}$	Entry of release matrix, defines whether microbial unit *i* releases compound unit *j*
$s=(s_j)$	Total supply of DOC from primary production
s_j	Supply of DOC compound unit *j* from primary production
$\boldsymbol{U}=(\boldsymbol{U}_{ij})$	Uptake matrix, defines which DOC compound units are taken up by which microbial unit
$\boldsymbol{U}_{ij}$	Entry of uptake matrix, defines whether microbial unit *i* takes up compound unit *j*

References

1. A. Ingalls, A. Pearson, Ten Years of Compound-Specific Radiocarbon Analysis. *Oceanography*. **18**, 18–31 (2005).
2. B. P. Koch, M. R. Witt, R. Engbrodt, T. Dittmar, G. Kattner, Molecular formulae of marine and terrigenous dissolved organic matter detected by electrospray ionization Fourier transform ion cyclotron resonance mass spectrometry. *Geochim. Cosmochim. Acta*. **69**, 3299–3308 (2005).
3. T. Dittmar, J. Paeng, A heat-induced molecular signature in marine dissolved organic matter. *Nat. Geosci.* **2**, 175–179 (2009).
4. J. M. Arrieta *et al.*, Dilution limits dissolved organic carbon utilization in the deep ocean. *Science (80-.).* **348**, 331–333 (2015).
5. Y. Shen, R. Benner, Mixing it up in the ocean carbon cycle and the removal of refractory dissolved organic carbon. *Sci. Rep.* **8**, 1–9 (2018).
6. N. Jiao *et al.*, Comment on: Dilution Limits Dissolved Organic Carbon Utilization in the Deep Ocean. *Science (80-.).* **350**, 1483a (2015).
7. O. A. Sosa, S. M. Gifford, D. J. Repeta, E. F. DeLong, High molecular weight dissolved organic matter enrichment selects for methylotrophs in dilution to extinction cultures. *ISME J.* **9**, 1–15 (2015).
8. D. A. Hansell, C. A. Carlson, D. J. Repeta, R. Schlitzer, Dissolved organic matter in the ocean. *Oceanography*. **22**, 202–211 (2009).
9. S. Roshan, T. DeVries, Efficient dissolved organic carbon production and export in the oligotrophic ocean. *Nat. Commun.* **8**, 2036 (2017).
10. D. A. Hansell, Recalcitrant Dissolved Organic Carbon Fractions. *Ann. Rev. Mar. Sci.* **5**, 421–445 (2013).
11. T. Dittmar, A. Stubbins, in *Treatise on Geochemistry*, H. D. Holland, K. K. Turekian, Eds. (Elsevier, Oxford, ed. 2nd, 2014), pp. 125–156.
12. N. Jiao *et al.*, Microbial production of recalcitrant dissolved organic matter: long-term carbon storage in the global ocean. *Nat. Rev. Microbiol.* **8**, 593–599 (2010).
13. B. P. Koch, G. Kattner, M. Witt, U. Passow, Molecular insights into the microbial formation of marine dissolved organic matter: Recalcitrant or labile? *Biogeosciences*. **11**, 4173–4190 (2014).
14. O. J. Lechtenfeld, N. Hertkorn, Y. Shen, M. Witt, R. Benner, Marine sequestration of carbon in bacterial metabolites. *Nat. Commun.* **6**, 6711 (2015).
15. H. Osterholz, J. Niggemann, H. A. Giebel, M. Simon, T. Dittmar, Inefficient microbial production of refractory dissolved organic matter in the ocean. *Nat. Commun.* **6** (2015), doi:10.1038/ncomms8422.
